# Viral targeting of TFIIB impairs *de novo* polymerase II recruitment and affects antiviral immunity

**DOI:** 10.1371/journal.ppat.1006980

**Published:** 2018-04-30

**Authors:** Darya A. Haas, Arno Meiler, Katharina Geiger, Carola Vogt, Ellen Preuss, Georg Kochs, Andreas Pichlmair

**Affiliations:** 1 Innate Immunity Laboratory, Max-Planck Institute of Biochemistry, Martinsried/Munich, Germany; 2 Institute of Virology, Medical Center—University of Freiburg, Freiburg, Germany; 3 Faculty of Medicine, University of Freiburg, Freiburg, Germany; 4 Technical University of Munich, School of Medicine, Institute of Virology, Munich, Germany; 5 German Center for Infection Research (DZIF), Munich partner site, Munich, Germany; Icahn School of Medicine at Mount Sinai, UNITED STATES

## Abstract

Viruses have evolved a plethora of mechanisms to target host antiviral responses. Here, we propose a yet uncharacterized mechanism of immune regulation by the orthomyxovirus Thogoto virus (THOV) ML protein through engaging general transcription factor TFIIB. ML generates a TFIIB depleted nuclear environment by re-localizing it into the cytoplasm. Although a broad effect on gene expression would be anticipated, ML expression, delivery of an ML-derived functional domain or experimental depletion of TFIIB only leads to altered expression of a limited number of genes. Our data indicate that TFIIB is critically important for the *de novo* recruitment of Pol II to promoter start sites and that TFIIB may not be required for regulated gene expression from paused promoters. Since many immune genes require *de novo* recruitment of Pol II, targeting of TFIIB by THOV represents a neat mechanism to affect immune responses while keeping other cellular transcriptional activities intact. Thus, interference with TFIIB activity may be a favourable site for therapeutic intervention to control undesirable inflammation.

## Author summary

Viruses target the innate immune system at critical vulnerability points. Here we show that the orthomyxovirus Thogoto virus impairs activity of general transcription factor IIB (TFIIB). Surprisingly, impairment of TFIIB function does not result in a general inhibition of transcription but in a rather specific impairment of selective genes. Transcriptome and functional analyses intersected with published CHIP-Seq datasets suggest that affected genes require *de novo* recruitment of the polymerase complex. Since the innate immune system heavily relies on genes that require *de novo* recruitment of the polymerase complex, targeting of TFIIB represents a neat mechanism to broadly affect antiviral immunity. Conversely, therapeutic targeting of TFIIB may represent a mechanism to limit pathological side effects caused by overshooting immune reactions.

## Introduction

Transcription of DNA by RNA polymerase II (Pol II) is central to gene expression and subject to regulation at multiple levels. A large number of dedicated protein complexes, as well as chromatin remodelling factors, are essential for transcription and regulate distinct phases of the transcriptional process [1]. The first step includes subunits of Pol II and general transcription factors (GTFs), which sequentially assemble into a preinitiation complex (PIC) that recognizes promoter regions on DNA and responds to regulatory signals in order to start mRNA synthesis [2]. GTFs include Transcription Factor (TF) IIA, TFIIB, TFIID, TFIIE, TFIIF, and TFIIH [3]. TFIID nucleates PIC assembly by binding promoter sequences through its TATA binding protein (TBP subunit) [4] and recruits TFIIB, which mediates the association of Pol II with the promoter [5]. Subsequently, TFIIE and TFIIH unwind DNA at the promoter for the initiation of transcription [6]. Pol II is released from the promoter to enable downstream transcription, while most of the GTFs dissociate from the promoter [7].

The recruitment of Pol II to the promoter is considered to be the major controlling step in gene expression [8]. However, an increasing number of genes are discovered to have higher accumulation of Pol II on their promoters without the corresponding enrichment within the gene bodies [9–12]. Such rate-limiting step, known as pausing, offers an additional regulatory switch in metazoans [13–17]. Pausing occurs when Pol II within the early elongation complex is prevented from further elongation through binding of Negative elongation factor (NELF) and DRB-sensitivity-inducing factor (DSIF) [18–20]. Release of paused Pol II is facilitated by positive transcription elongation factor (P-TEFb) enabling productive elongation and mRNA transcription [16,18,21].

Regulated gene expression is crucial for most biological processes. Particularly tight control of gene expression is required for controlling the immune system. Expression of immune-related genes is strictly regulated under non-perturbed conditions, while invasion of pathogens, such as viruses and bacteria, or toxins, induce a plethora of pro-inflammatory cytokines and expression of anti-viral/bacterial effector proteins. Virus-induced expression of the human interferon (IFN)-β gene is one of the best characterized examples for inducible gene expression in higher eukaryotes. IFN-β is not expressed under steady state conditions but is synthesized in response to virus infection. This process relies on the sensing of virus infection or replication products by dedicated pattern recognition receptors (PRRs), which initiate a signalling cascade that leads to local disassembly of nucleosomes and activation of transcription factors including interferon regulatory factors (IRF) 3 and -7 [22,23], which are master regulators of type-I IFNs (IFN-α/β). Secreted IFN-α/β bind type-I IFN receptor [24], which trigger signal transducer and activator of transcription (STAT)-1 and -2 [25], which lead to the transcription of several hundred genes encoding proteins with antiviral properties or regulatory functions mounting an efficient antiviral barrier.

Most viruses evolved mechanisms to target vulnerable points of the antiviral defence system. These mechanisms often include viral perturbations of transcriptional processes. However, viral strategies vary widely: while some large DNA viruses such as pox- and herpesviruses express a number of open reading frames (ORFs) acting in parallel to impair sensing, signalling and activation of transcription factors [26–31], small RNA viruses with limited genetic coding capacity preferentially perturb the antiviral system at critical hubs. The Npro protein encoded by the positive strand RNA virus Bovine viral diarrhoea virus (BVDV), for instance, specifically leads to proteasomal degradation of IRF3 and thus alleviates induction of IFN-α/β [32,33]. Rift Valley fever virus (RVFV), a negative strand RNA virus belonging to the virus family of Bunyaviridae, degrades polymerase II subunit TFIIH through its Non-structural protein Small (NSs) [34,35] resulting in general inhibition of transcription, followed by broad inhibition of protein expression and detrimental effects for cell viability. An orthomyxovirus Thogoto virus (THOV) also targets the polymerase complex through association of its Matrix protein long (ML) with TFIIB [36]. However, in contrast to RVFV NSs, THOV ML expression appears not to lead to general inhibition of gene expression and plasmid-driven ML expression does not show an apparent toxicity effect. Instead, it is believed that ML specifically impairs expression of type-I interferon genes through engaging IRF3 [37–40].

However, several lines of evidence suggest an activity of ML exceeding simple inhibition of IRF3. These include inability of THOV ML to inhibit IRF3 nuclear translocation [38]. Unbiased evaluation of THOV ML interacting proteins by affinity purification coupled with mass spectrometry (AP-MS) as well as *in vitro* binding assays (data shown here and [40]) did not support direct interaction of THOV ML with IRF3. Furthermore, ML has also been proposed to impair expression of NF-kappa B target genes [40], which can be induced independently of IRF3 [41]. We therefore asked how ML can display an exceptionally specific response, by targeting a very central transcription factor. Moreover, we expected to expand our knowledge on the functionality of TFIIB in higher eukaryotes.

## Results

### Thogoto virus ML protein inhibits IFN-α/β production by interacting with TFIIB

**Fig 1:**
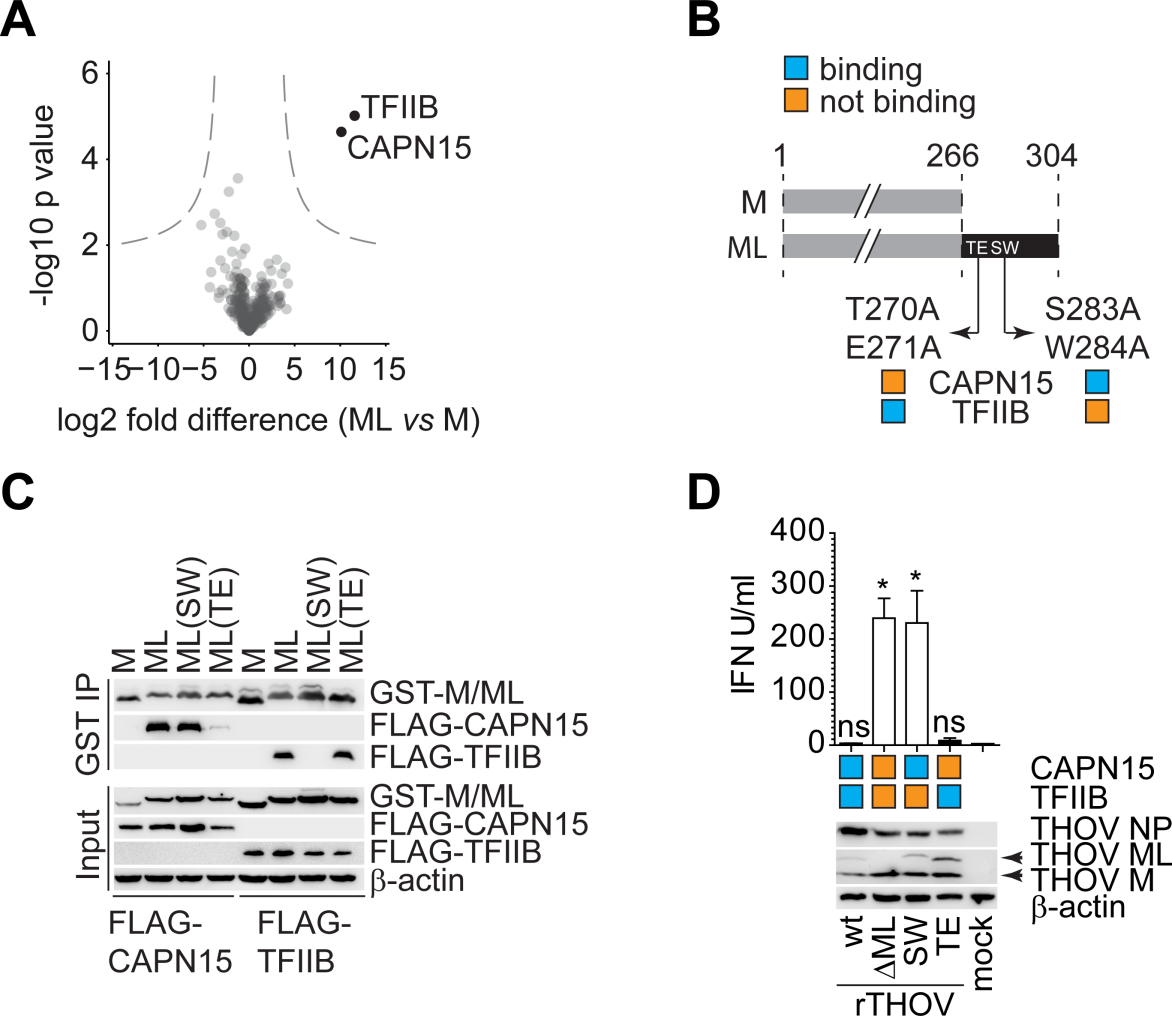
Thogoto virus ML protein inhibits IFN-α/β production by interacting with TFIIB. A) Volcano plot of proteins enriched in ML vs. M pulldown in HEK293 cells and identified by AP-LC-MS/MS. HA-tagged M or ML proteins were overexpressed in 4 biological replicates. B) Schematic representation of ML protein and its mutants not binding TFIIB or CAPN15. C) IP of GST-tagged M or ML (wt and mut) and co-IP of FLAG-tagged CAPN15 and TFIIB transiently overexpressed in HEK293 cells. Western blot is a representative of two independent experiments with similar results. D) IFN-α/β levels after infection with THOV wt, ΔML or mutant ML(SW) 24 h.p.i. SN from infected HeLa cells were applied to 293T Mx1-luc. *—p value < 0.05, NS–non-significant. Bar graph shows mean with SD of three technical replicates and is a representative of four independent experiments with similar results. Significance was estimated with Kruskal-Wallis test with Dunn’s multiple comparison post-test.

To identify proteins specifically involved in IFN-α/β inhibition by Thogoto virus (THOV), we used affinity purification followed by liquid chromatography and tandem mass spectrometry (AP-LC-MS/MS). To this end, we expressed HA-tagged viral M and ML proteins in HEK293 cells and performed affinity purification followed by LC-MS/MS analysis. In line with the large shared regions between both proteins, the majority of identified proteins were equally well enriched in M and ML precipitates (Fig 1A and 1B, S1A Fig, S1 Table). However, we identified two interactors that were highly significantly enriched in ML samples: the general transcription factor IIB (TFIIB) and the largely uncharacterized CAPN15, a member of the calpain family of proteases (Figs 1A and S1A).

We validated ML-specific binding of GST-tagged M and ML to FLAG-tagged CAPN15 and TFIIB (Fig 1C). In order to refine binding requirements we also used C-terminal point mutants of ML proteins bearing alanine replacements at positions S283A/W284A (SW) and T270A/E271A (TE) (Fig 1B). As published previously[40], ML(SW) lost the ability to interact with TFIIB, whereas ML(TE) bound to TFIIB similarly well to the wild type protein. Conversely, we found that CAPN15 only inefficiently precipitated with exogenously expressed ML(TE) but bound TFIIB comparably well to the wt ML protein (Fig 1B and 1C). To validate these findings in an infection context, we generated recombinant THOV that expressed corresponding mutants of ML. As expected, THOV expressing ML(SW) lost the ability to interact with TFIIB while it retained binding to CAPN15 and virus expressing ML(TE) lost binding to CAPN15 while it bound to TFIIB (S1B Fig). Infection of HeLa cells with recombinant THOV wild type (rTHOV-wt) as well as with the virus bearing TE mutation in ML (rTHOV-TE), which is still capable to bind TFIIB but cannot bind CAPN15, induced only minimal amounts of IFN-α/β (Fig 1D). In contrast, ML deletion mutant rTHOV-ΔML and SW ML (rTHOV-SW), which lost binding to TFIIB but retained CAPN15 affinity, potently stimulated IFN-α/β production (Fig 1D). From these experiments we concluded that the ability of ML to interact with TFIIB is necessary and sufficient to interfere with IFN-α/β induction and that binding to CAPN15 does not affect induction of type-I interferons.

### ML sequesters TFIIB from the nucleus, but does not cause its degradation

**Fig 2:**
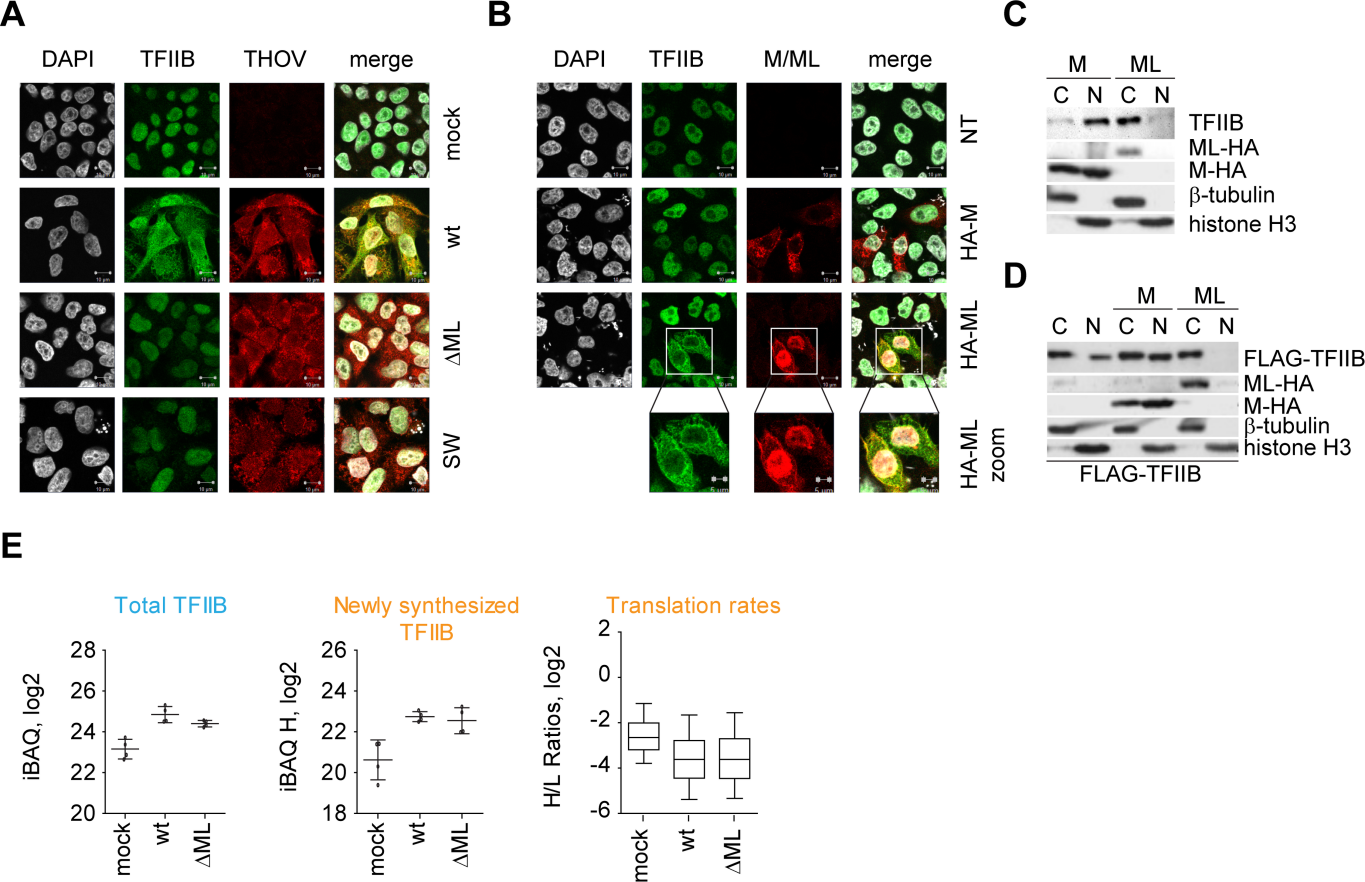
ML sequesters TFIIB from the nucleus, but does not cause its degradation. A) Confocal immunofluorescence analysis of HeLa Kyoto cells stably expressing GFP-TFIIB infected with THOV wt, THOV-ΔML or THOV-SW mutants for 24 hours at MOI 3. HeLa cells were treated as indicated, fixed and stained with GFP-DyLight488, THOV NP+rbAlexa546 and DAPI and subjected to confocal microscopy. Images are representative of two independent experiments with similar results. White bar – 10 μm. B) Confocal immunofluorescence analysis of HeLa Kyoto cells stably expressing GFP-TFIIB and transiently transfected with HA-M or HA-ML for 16 hours. HeLa cells were treated as indicated, fixed and stained with GFP-DyLight488, HA+msAlexa594 and DAPI and subjected to confocal microscopy. Images are representative of four independent experiments with similar results. White bar – 10 μm. C) Cytoplasmic-nuclear fractionation of Vero cells transiently transfected with HA-tagged M or ML for 24 hours. Depicted is endogenous TFIIB. Western blot is a representative of three experiments with similar results. D) Cytoplasmic-nuclear fractionation of Vero cells transiently transfected with FLAG-TFIIB and HA-tagged M or ML for 24 hours. Western blot is a representative of three experiments with similar results. E) Total protein intensity of TFIIB as defined by iBAQ, levels of newly synthesized TFIIB as determined from heavy intensities, and translation rates of identified proteins determined from H (newly synthesized)/L (total) ratios presented as box-whisker plots with whiskers showing 10–90 percentile. Protein levels were estimated in four biological replicates.

To further understand how ML interferes with the function of TFIIB, we tested distribution of TFIIB on a subcellular level by confocal imaging. In uninfected cells TFIIB showed nuclear localization (Fig 2A), consistent with its function in RNA transcription [42]. Surprisingly, rTHOV-wt infected cells showed a dramatic re-localization of TFIIB from the nucleus into the cytoplasm (Fig 2A). This re-localization was specific to the ability of ML to bind TFIIB, since infection with recombinant THOVs that expressed ML variants with impaired TFIIB binding, such as rTHOV-ΔML and rTHOV-SW, resulted in nuclear TFIIB localization (Fig 2A). To test whether the observed effect was indeed dependent on ML activity, we transiently transfected HA-tagged ML and analyzed subcellular localization of TFIIB. In line with infection experiments, transfection of ML, but not M, led to accumulation of TFIIB in the cytoplasm already at 16 h.p.t. (Fig 2B), where it also remained at 24 h.p.t. (S2A Fig). Additionally, we could confirm TFIIB re-localization by subcellular fractionation of ML transfected cells. In cells expressing M, endogenous (Fig 2C), as well as overexpressed (Fig 2D) TFIIB localized to the nucleus, whereas in cells that received ML TFIIB was almost exclusively detectable in the cytoplasm (Figs 2C and 2D). Of note, ML did not generally affect nuclear/cytoplasmic distribution since histone H3 still localized to the nucleus (Figs 2C and 2D). Altogether, these data suggested that ML interferes with TFIIB function by altering its subcellular distribution and therefore would not allow TFIIB to contribute to transcriptional processes.

It is known that other viruses similarly interact with components of transcription machinery. RVFV NSs protein, for instance, sequesters the p44 subunit of TFIIH [43] and recruits an E3 ligase complex to the p62 subunit leading to its proteasomal degradation [35] resulting in destabilization of TFIIH and general shutdown of mRNA transcription. To test whether ML impairs the stability of TFIIB, we employed a pulse-SILAC LC-MS/MS approach that allowed us to assess proteome-wide protein abundance, stability and translation rates of 5416 proteins (S2B Fig, S2 Table). Compared to mock, infection with both rTHOV-wt and rTHOV-ΔML slightly increased total and newly synthesized TFIIB levels (Fig 2E). However, there was no difference between rTHOV-wt and rTHOV-ΔML (Fig 2E). The expression and stability of other PIC components were not affected by infection with rTHOV-wt either (S2C Fig). Moreover, although the total levels of protein translation were reduced in virus infected cells, no difference between rTHOV-wt or rTHOV-ΔML infection could be seen (Fig 2E). Collectively, these data suggested that ML binds to TFIIB but this interaction does not lead to generally reduced abundance of TFIIB or PIC proteins and does not affect general translation rates in THOV infected cells.

### Transcriptome analysis of THOV-induced changes suggests broad but selective effect of ML

**Fig 3:**
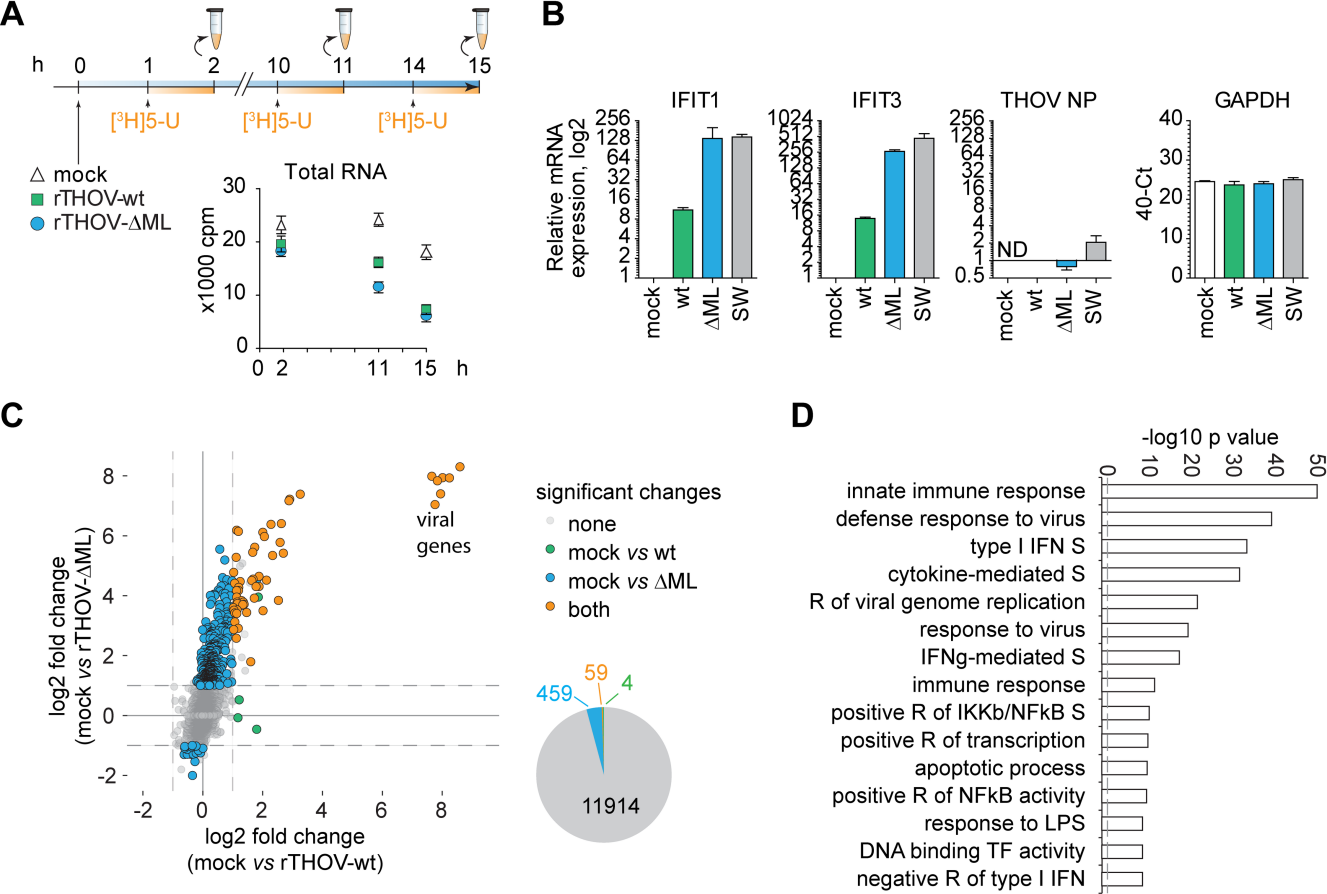
Transcriptome analysis of THOV-induced changes suggests broad but selective effect of ML. A) RNA synthesis rate in THOV infected Vero cells. Newly synthesized RNA was labelled with [3H]-5-Uridine at 1, 10 and 14 hours post infection. Presented is total RNA fraction as mean and SD from three technical replicates. B) qPCR analysis of IFITs, THOV NP transcript and GAPDH expression in HeLa cells after infection with THOV-wt, THOV-ΔML and THOV-SW for 16 hours. Presented are means and SD from three independent infection experiments. C) 2D-scatter plot of transcriptome analysis of HeLa cells infected with THOV-wt, THOV-ΔML and THOV-SW for 16 hours in three biological replicates. X axis represents log2 fold changes between mock and THOV-wt (green dots), Y axis shows log2 fold changes between mock and THOV-ΔML (blue dots). Changes occurring in wt and ΔML infections are shown in orange. Unchanged genes are shown in grey. Numbers are presented in a pie chart. Differentially regulated genes were showing at least 2-fold change with a q value < 0.05. D) GO term over-representation analysis of differentially upregulated genes by THOV ΔML and THOV ML (SW/AA) compared to THOV wt performed with InnateDB analysis tool. S–signalling, R–regulation, TF–transcription factor.

Based on the ability of ML to impair cellular localization of a central component of the PIC, we examined whether ML has a broader effect on transcription than previously anticipated. To this aim, we performed pulse-labelling experiments using radioactively labelled [^3^H]5-Uridine (Fig 3A), which is incorporated into newly synthesized RNA and therefore allows to assess global transcription rates in a time-dependent manner. In agreement with the levels of translation, total RNA synthesis was similar in rTHOV-wt and rTHOV-ΔML infected cells (Fig 3A).

To test the effect of ML in an unbiased manner, we performed transcriptome analysis of HeLa cells that were left uninfected or infected with rTHOV-wt, rTHOV-ΔML or rTHOV-SW (S3A Fig, S3 Table, GEO GSE105152). The RNA levels of viral NP transcripts were comparable in virus infected cells and not detected in mock (Fig 3B). As expected, infection with rTHOV-ΔML and rTHOV-SW elicited expression of IFIT1 and IFIT3, whereas their expression in rTHOV-wt sample was ~50 fold lower (Fig 3B). Interestingly, expression of housekeeping genes, such as GAPDH, appeared quite stable independently of the virus used (Fig 3B), validating the lack of general inhibition on the transcriptional level. Whole transcriptome analysis using next generation sequencing of cells infected with rTHOV-wt revealed only 63 significantly changed genes (q<0.05; fold change ≥2) (Fig 3C, x-axis, green and orange dots, S3 Table), which is in line with our above findings that targeting of TFIIB by ML does not significantly impact general transcription rates in a negative or positive manner. In contrast, in cells that were infected with rTHOV-ΔML we identified 518 differentially regulated genes compared to mock (q<0.05; fold change ≥2) (Fig 3C, y-axis, blue and orange dots, S3 Table). rTHOV-SW elicited a comparable response to rTHOV-ΔML infection, underlining that changes occurred in a TFIIB-dependent manner (S3B Fig, S3 Table). General properties of genes significantly upregulated by rTHOV-ΔML and rTHOV-SW (S3B Fig) were related to innate antiviral response as determined by GO term over-representation analysis (Fig 3D). Additionally, we screened promoter regions of co-regulated genes for putative transcription factor binding sites [44] to identify gene populations with diverse nature of regulation. This analysis suggested that compared to ML mutant viruses, the wt virus blunted expression of genes that were under control of diverse transcription factors. Most affected were IRFs and STATs, but also RELA, SPI1, and FOS (S3C Fig, S4 Table). In sum, these data suggested that general transcription rates and transcription of constitutively active genes were not affected by THOV infection and that the wild-type virus inhibited a broad array of inducible inflammation-related and -unrelated genes driven by diverse transcription factors.

### Multiple but not all inducible promoters are repressed by ML

**Fig 4:**
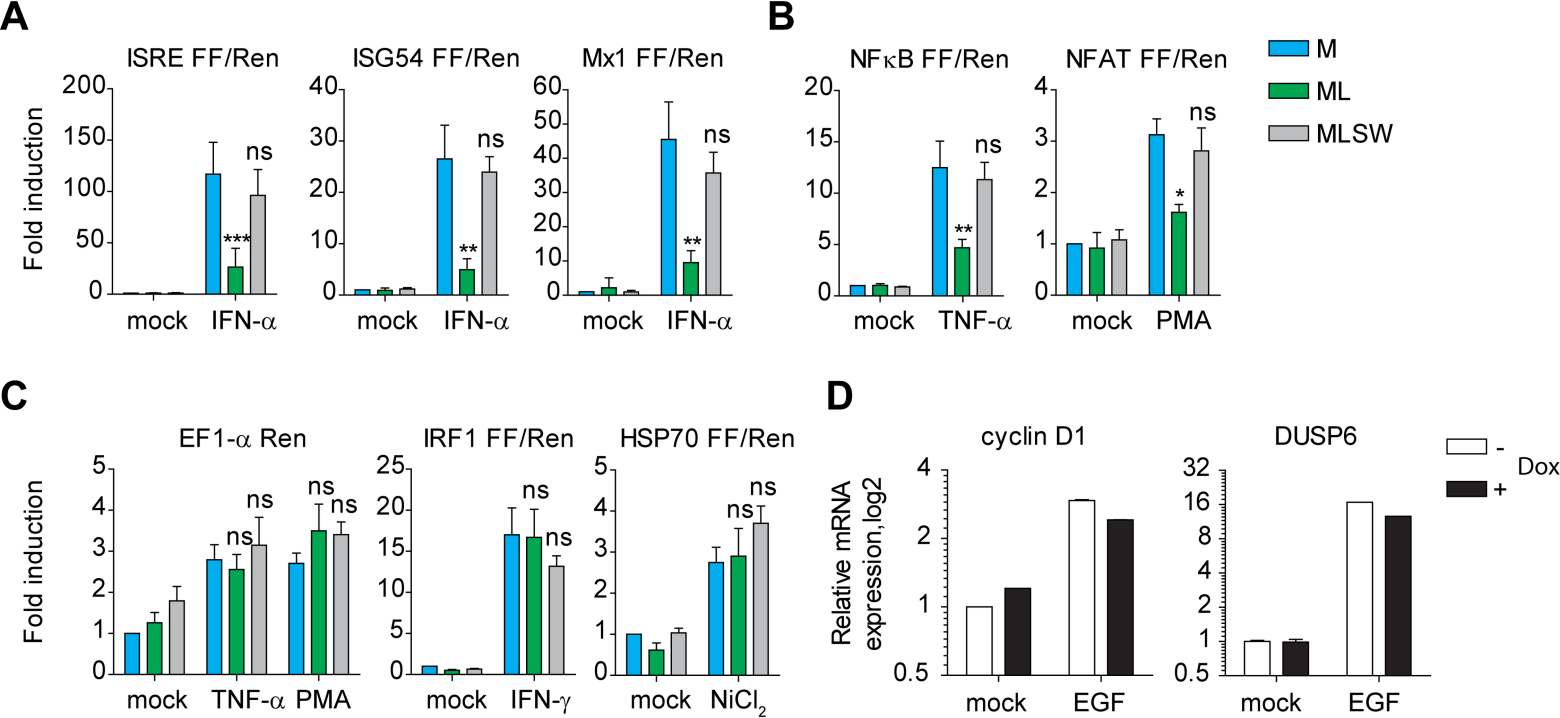
Multiple but not all inducible promoters are repressed by ML. (A-C) Reporter assays in HEK293 cells, where Firefly luciferase under indicated promoters was transfected together with EF-1a Renilla and M/ML/ML-SW. 24 h.p.t. cells were treated with indicated stimuli for 16 hours and the luciferase activity was measured. Shown is fold induction (mean and SD) over untreated cells from three independent experiments performed in six technical replicates. *—p<0.05; **—p<0.01; ***—p<0.001; ns–not significant. D) qPCR analysis of HeLa FlipIn cells expressing stably integrated ML from Tet-On promoter. HeLa cells were left untreated or treated with Doxycycline for 24h and subsequently stimulated with EGF for 16 hours. Total RNA was extracted and expression of indicated genes measured by qPCR. Shown are expression levels in relation to the non-Doxycycline treated unstimulated condition. The bar graphs show mean +/- error from two technical replicates and are representative of two independent experiments with similar results.

Transcriptome profiling suggested that cells infected with rTHOV-wt show minimal changes in gene expression compared to mock infected cells. This prompted us to test, whether ML may selectively inhibit any dynamic changes in gene expression without affecting ongoing transcriptional processes. To test this and to delineate whether ML is not just inhibiting upstream processes resulting in broad spectrum of changes, we employed reporter constructs that allow to directly measure the transcriptional activity of selected promoters after stimulation with defined activating ligands. We used type-I IFN driven luciferase reporter constructs such as promoters for interferon stimulated response element (ISRE), the ISG54 promoter or Mx1, all of which contain STAT1 binding sites and thus are directly responsive to IFN-α/β treatment. As expected, co-expression of ML impaired their activity, whereas M or ML(SW) did not show this effect (Figs 4A and S4A).

The inhibitory effect of ML, but not M or ML(SW) could also be seen for other inducible promoters containing NFκB or NFAT sites (Figs 4B and S4B). However, in agreement with minimal influence on constitutive gene expression, the activity of EF1-α-promoter-driven Renilla luciferase was not affected by co-transfection of M, ML or ML(SW) (Figs 4C and S4C). Surprisingly, IRF1 promoter, even though showing a dynamic change after IFN-γ stimulation, was not affected by ML overexpression (Figs 4C and S4C). Similarly, HSP70 promoter (Figs 4C and S4C) and EGF targets cyclin D1 and DUSP6 were induced equally well despite ML presence (Figs 4D and S4D). From these experiments we concluded that ML represses dynamic changes in gene expression, but this inhibitory effect is limited to the activity and regulation of selected promoters.

### TFIIB depletion preferentially affects a subset of inflammatory cytokines and antiviral effector genes

**Fig 5:**
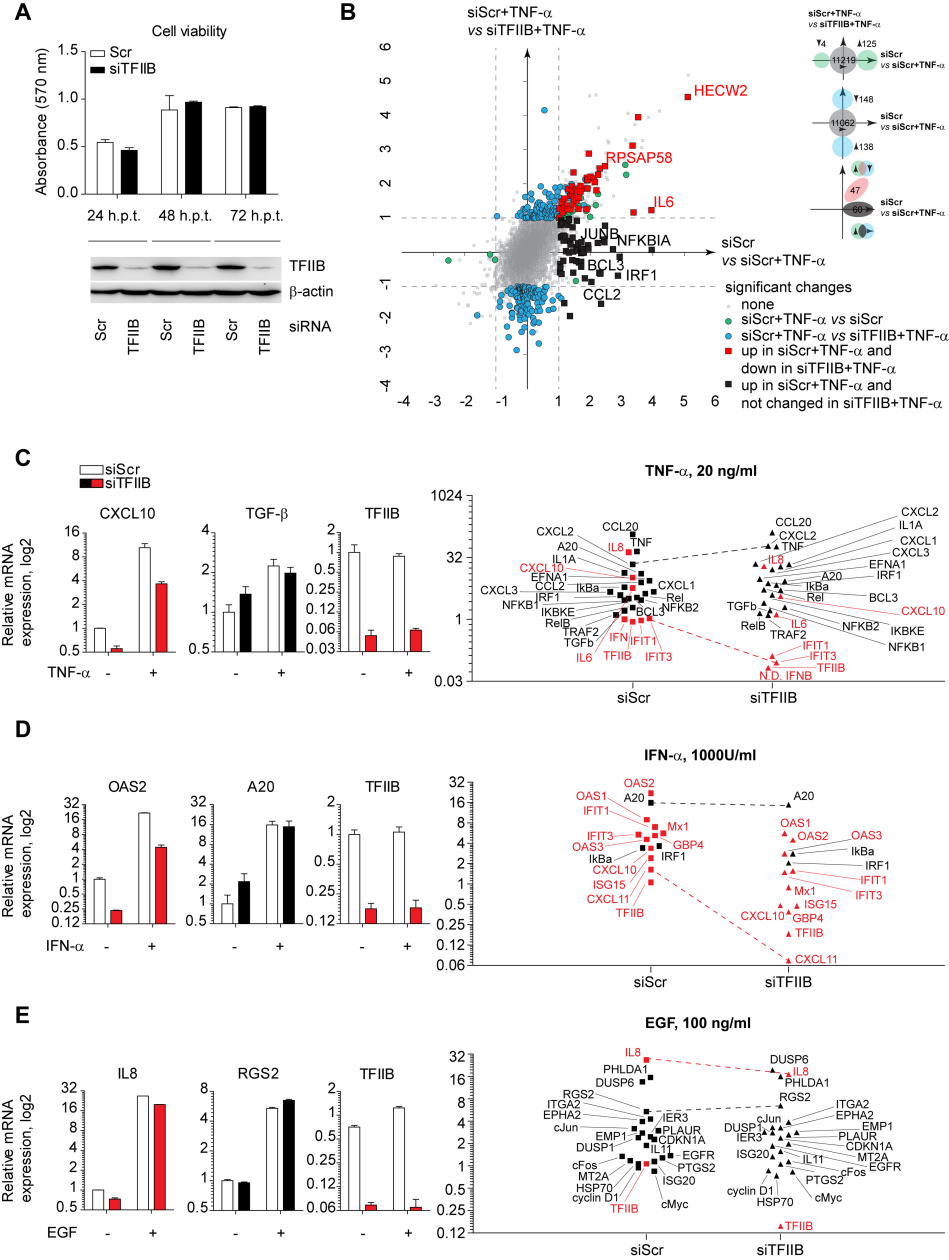
TFIIB depletion preferentially affects a subset of inflammatory cytokines and antiviral effector genes. A) Viability of HeLa cells after TFIIB knockdown at indicated time points. HeLa cells were treated with indicated siRNAs for 24, 48 and 72 hours and knockdown was validated by Western blot analysis. Cell viability was assessed by MTT assay. The bar graph shows mean and SD of three technical replicates and is a representative of two independent experiments with similar results. B) 2D-scatter plot of transcriptome analysis of HeLa cells before and after TFIIB knockdown mock-treated or stimulated with TNF-α in three biological replicates. HeLa cells were electroporated with Scrambled or TFIIB-targeting siRNAs. In 24 hours they were left untreated or stimulated with TNF-α (20 ng/ml) for 2 hours. Total RNA was extracted and analysed by RNA-seq. X axis shows log2 fold changes between mock-treated and TNF-α-treated cells with non-targeting siRNA (green dots), Y axis represents log2 fold changes between TNF-α-treated Scrambled-transfected cells and TNF-treated siTFIIB-transfected cells (blue dots). Genes upregulated by TNF-α in non-targeted cells and downregulated by TFIIB knockdown are shown in red. Genes upregulated by TNF-α in non-targeted cells and not regulated by TFIIB knockdown are shown in black. Numbers are shown in the scheme. Differentially regulated genes were showing at least 2-fold change with a q value < 0.05. C) qPCR analysis of genes regulated by TNF-α stimulation and affected (red) or non-affected (black) by TFIIB knockdown in HeLa cells. Bar graphs show representative genes. Scatter plots show all genes analysed. Dashed lines are showing the direction of change for affected (red) and non-affected (black) genes. Log2 fold changes are shown relative to Scrambled-transfected mock-treated cells. D) qPCR analysis of genes regulated by IFN-α stimulation. E) qPCR analysis of genes regulated by EGF stimulation. For all stimuli HeLa cells were electroporated with indicated siRNAs and in 16 hours stimulated with indicated ligands for 4 hours with subsequent RNA extraction. qPCR analysis was performed in two technical replicates in two independent experiments.

Since ML affects gene expression by interacting with TFIIB, we hypothesized that TFIIB may be required for expression of selected genes and that depletion of TFIIB would mimic the effect of ML. To test this, we performed siRNA-mediated knockdown of TFIIB and tested whether this influenced gene expression from inducible promoters. Transient depletion of TFIIB did not affect cell viability within the timeframe of this experiment (Fig 5A), which is in line with a recent report[45].

In order to gain insights into genome-wide distribution of TFIIB-dependent genes, we performed RNA-seq analysis after TFIIB depletion followed by stimulation with TNF-α (S5A Fig). This experimental setup allowed us to compare the impact of TFIIB depletion on general gene expression as well as the cellular response to a defined stimulus (TNF-α treatment) (Fig 5B, S5 Table, GEO GSE105153). In control siRNA-treated cells, TNF-α treatment induced expression of 125 genes (Fig 5B, x-axis, green dots). Compared to control knockdown, TFIIB depletion in combination with TNF-α treatment led to a minor difference in the overall gene expression pattern–from 11348 mapped genes only 148 genes showed decreased and 138 genes increased expression (Fig 5B, y-axis, blue dots). Notably, TFIIB depletion resulted in two populations of TNF-α responsive genes: 47 TNF-induced genes were inhibited more than 2-fold in expression by TFIIB depletion (Fig 5B, red squares), while 60 genes were induced similarly in TFIIB depleted and control cells (Fig 5B, black squares). We validated this effect by qPCR for two exemplary targets: IFN-β expression was severely affected by TFIIB depletion while induction of the control gene Ephrin-A1 (EFNA1) was comparable in control and TFIIB depleted cells (S5B Fig). We performed qRT-PCR on independent samples applying three distinct stimuli, TNF-α, IFN-α and EGF, all of which induce a specific gene expression profile (S6 Table). As expected TNF-α led to two distinct populations of genes: genes that showed ≥1.5-log2 fold decreased expression in the absence of TFIIB (Fig 5C, red symbols) and genes, which were equally induced despite TFIIB depletion (Fig 5C, black symbols). In agreement with RNA-seq analysis, TFIIB was required for the activation of inflammatory cytokines IL-6, IL-8, CXCL10 and IFITs (Fig 5C, red symbols), whereas induction of EFNA1, IKBKE, IRF1, A20 and IκBα was not significantly altered (Fig 5C, black symbols). TFIIB was also required for inducible expression of some IFN-α/β stimulated targets: IFN-α/β dependent induction of OAS2, GBP4, CXCL10, CXCL11, ISG15, Mx1 and IFITs was significantly impaired in the absence of TFIIB (Fig 5D, red symbols). However, IFN-α stimulation of other genes such as A20, IRF1, IκBα was not affected by TFIIB depletion (Fig 5D, black symbols). In line with minimal effects of ML on EGF targets (Fig 4D), direct TFIIB knockdown did not significantly affect basal or induced expression of any of the EGF responsive gene tested, besides IL-8 (Fig 5E). Similarly, TFIIB depletion after infection with rTHOV-ΔML (mixed stimulus) led to a decreased IFIT3 expression, but not STAT1, p65 or p38 (S5C Fig). Overall, despite different ligands that had been used for stimulation, components of signalling pathways, were induced comparably well in siTFIIB and siScrambled treated cells. In contrast, expression of inflammatory cytokines and genes encoding antiviral effector proteins was severely impaired (Fig 5C–5E).

### Genes subject to TFIIB regulation require *de novo* Pol II recruitment

**Fig 6:**
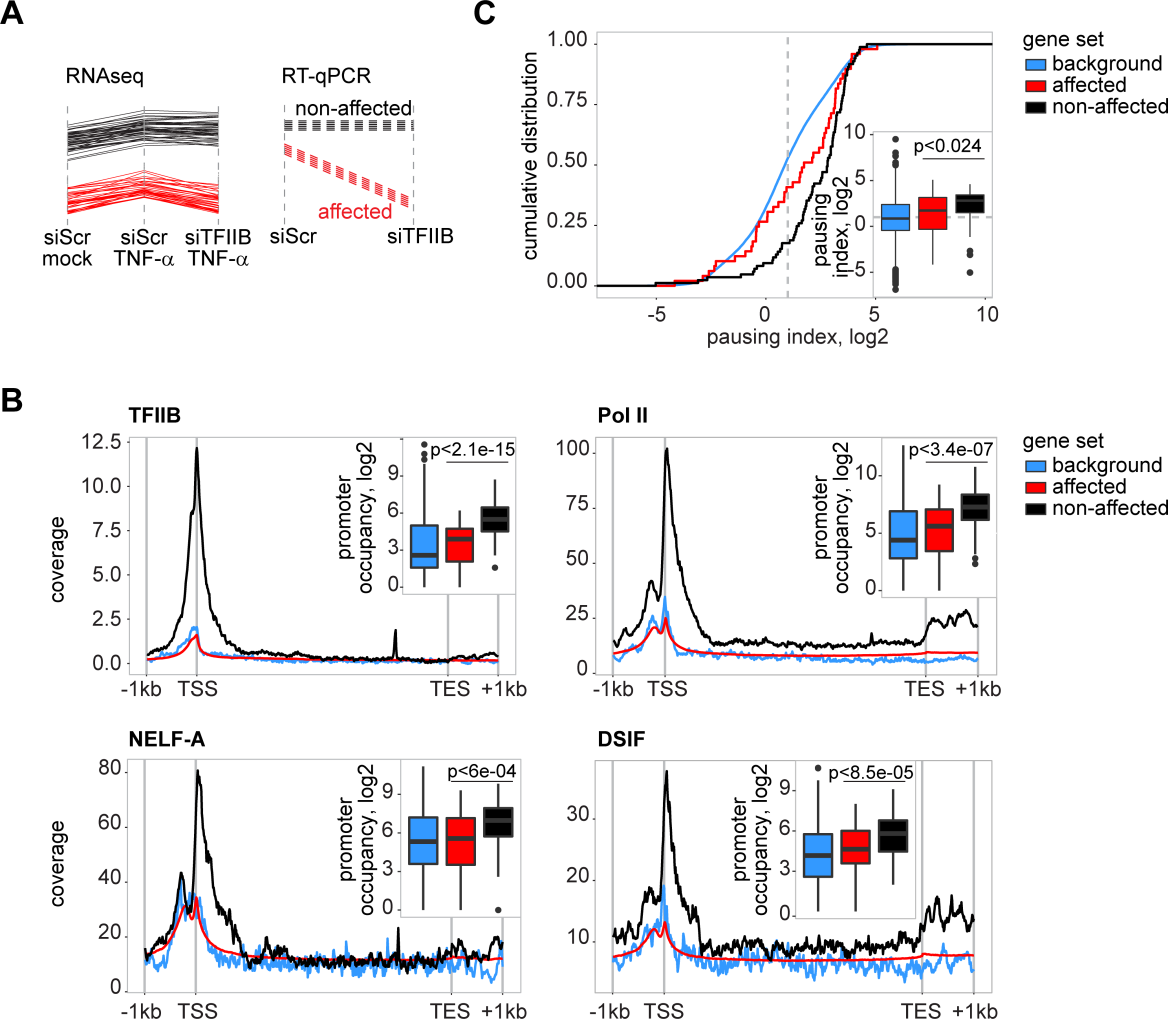
Genes subject to TFIIB regulation require *de novo* Pol II recruitment. A) Schematic representation of profile plots of the genes affected (red) and non-affected (black) by TFIIB knockdown in RNA-seq (top panel) and qPCR screen (bottom panel). B) Gene-averaged occupancy profiles of TFIIB, Pol II, NELF-A and DSIF for genes affected (red) or non-affected (black) by TFIIB depletion and for the genome background (blue). Embedded boxplots show the distribution of ChIP-Seq read coverage in the downstream promoter (Pol II, NELF, DSIF) or promoter (TFIIB) regions across genes. C) Distribution of pausing indices (pi) between affected and non-affected genes (pi>2 can be considered paused).

In general, two different modes of transcriptional regulation exist: (i) gene expression can be regulated through pausing of already recruited Pol II at the promoter when stimulus-dependent activation releases this pausing activity to drive expression of genes (S6A Fig). Prototypic genes falling in this category of gene regulation are targets of the EGF signalling cascade [8,46,47]. (ii) Another mechanism to regulate gene expression is *de novo* recruitment of Pol II to transcriptional start sites (S6A Fig). This mode of regulation is best described for a subset, but not all genes associated with inflammatory processes [48]. The fact that EGF targets were not regulated by TFIIB depletion (Figs 4D and 5E) could be attributed to their regulation by polymerase pausing [46], which potentially does not require TFIIB. The diverse behaviour of signalling components and inflammatory cytokines in respect to TFIIB knockdown (Fig 5B–5E) further suggested *de novo* Pol II recruitment as the main discriminating feature of the affected genes. To corroborate this observation, we performed analysis of publically available chromatin-immunoprecipitation followed by deep sequencing (ChIP-Seq) datasets and calculated the occupancies of Pol II, TFIIB, NELF and DSIF [49,50] at the promoter and gene body regions (Fig 6, S7 Table).

These datasets allowed us to define genes that are actively transcribed (high Pol II in the gene body), paused (high Pol II at the promoter and low in the gene body, high NELF and DSIF at the promoter) or not occupied by polymerase or pausing-associated factors and therefore require *de novo* recruitment of the pre-initiation complex (low Pol II at the promoter and in the gene body, low NELF and DSIF at the promoter) (S6 Fig). The status of TFIIB during elongation and pausing is not well characterized, thus we speculated that TFIIB levels at the promoters would be reflective of those of Pol II. We then mapped identified occupancies to the genes, found to be influenced by TFIIB depletion in RNA-seq experiments (Figs 5B and 6A red profiles) and the qPCR screen (Figs 5C–5E and 6A red dashed lines), and those genes observed to be non-affected by TFIIB depletion (Figs 5B–5E and 6A black profiles). Remarkably, genes affected by TFIIB depletion showed significantly lower occupancies of Pol II, and TFIIB in the promoter region, as compared to non-affected genes (Fig 6B). In addition, promoters of genes that were not affected by TFIIB depletion, showed high occupancies of NELF and DSIF, which are markers for paused promoters. Indeed, calculating pausing indices (promoter and gene body occupancy ratio > 2) revealed significantly higher pausing rates in non-affected vs affected genes (Fig 6C), demonstrating that non-affected genes are regulated by promoter-proximal pausing and supporting our hypothesis of *de novo* recruitment as mechanistic requirement for regulation of genes affected by TFIIB depletion. In sum, these data indicate that TFIIB is needed for induced expression of genes that require *de novo* polymerase II recruitment and that TFIIB absence is better tolerated by genes regulated through polymerase pausing. Furthermore, these data are consistent with the inhibitory activity of ML on reporter constructs and explain the broad, yet relatively selective activity of wild-type THOV.

### A minimal ML sequence required for TFIIB inhibition

**Fig 7:**
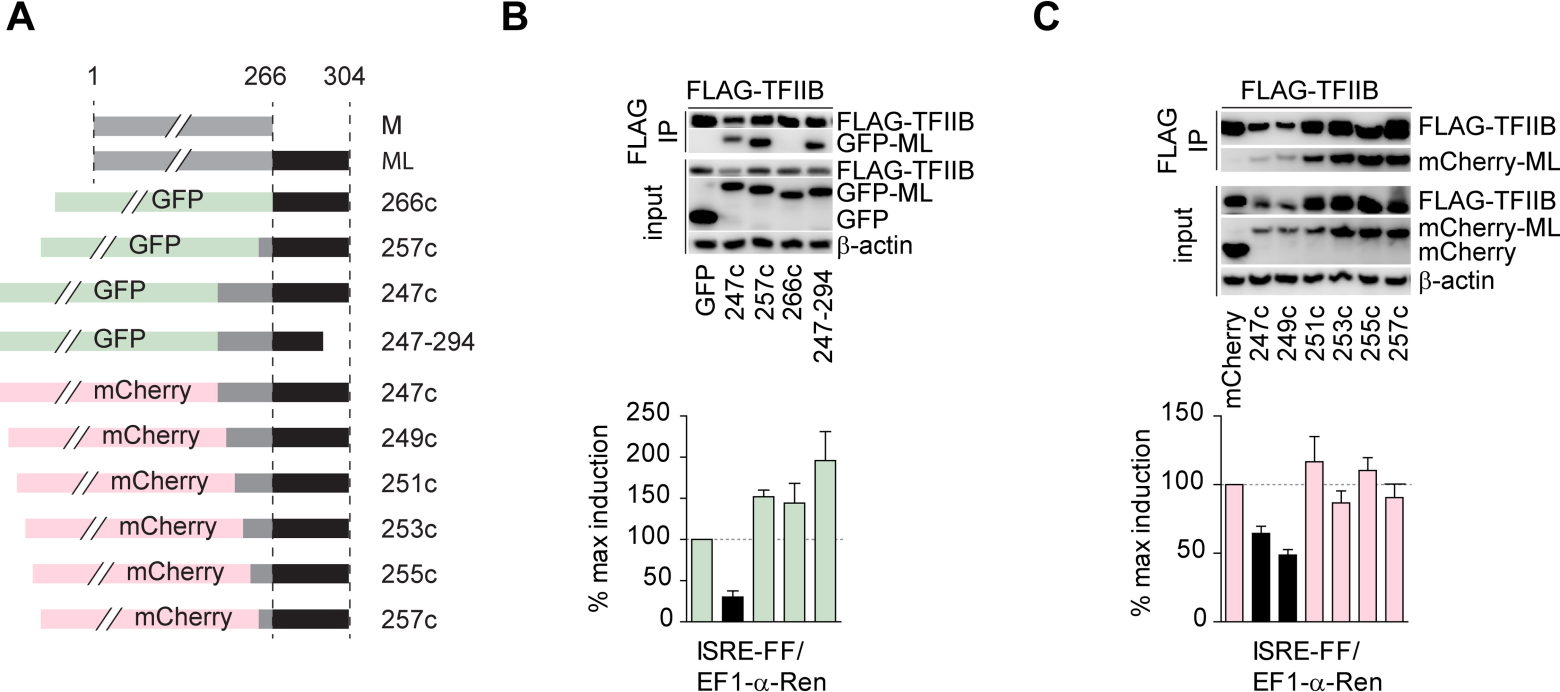
Mapping of a minimal ML sequence required for TFIIB inhibition. A) Schematic representation of ML fragments. B) Top panel: IP of FLAG-tagged TFIIB and GFP-fused ML fragments. Bottom panel: reporter assay in HEK293 cells, where Firefly luciferase under ISRE promoter was co-transfected with EF1-α-Renilla and GFP-ML fragments. C) Top-panel: IP of FLAG-tagged TFIIB and mCherry-fused ML fragments. Bottom panel: reporter assay in HEK293 cells, where Firefly luciferase under ISRE promoter was co-transfected with EF1-α-Renilla and mCherry-ML fragments. Western blots are representative of two experiments with similar results. Bar graphs show mean and SD from three technical replicates and are representative of two experiments with similar results.

Selective regulation of gene expression would allow for the possibility to interfere with *de novo* transcription, which would be of particular interest to modulate inflammatory processes associated with pathogenicity and disease. Compared to M, ML has a 38 amino acids (aa) C-terminal extension (Fig 7A) and we hypothesized that expression of this fragment may be sufficient to regulate TFIIB activity.

**Fig 8:**
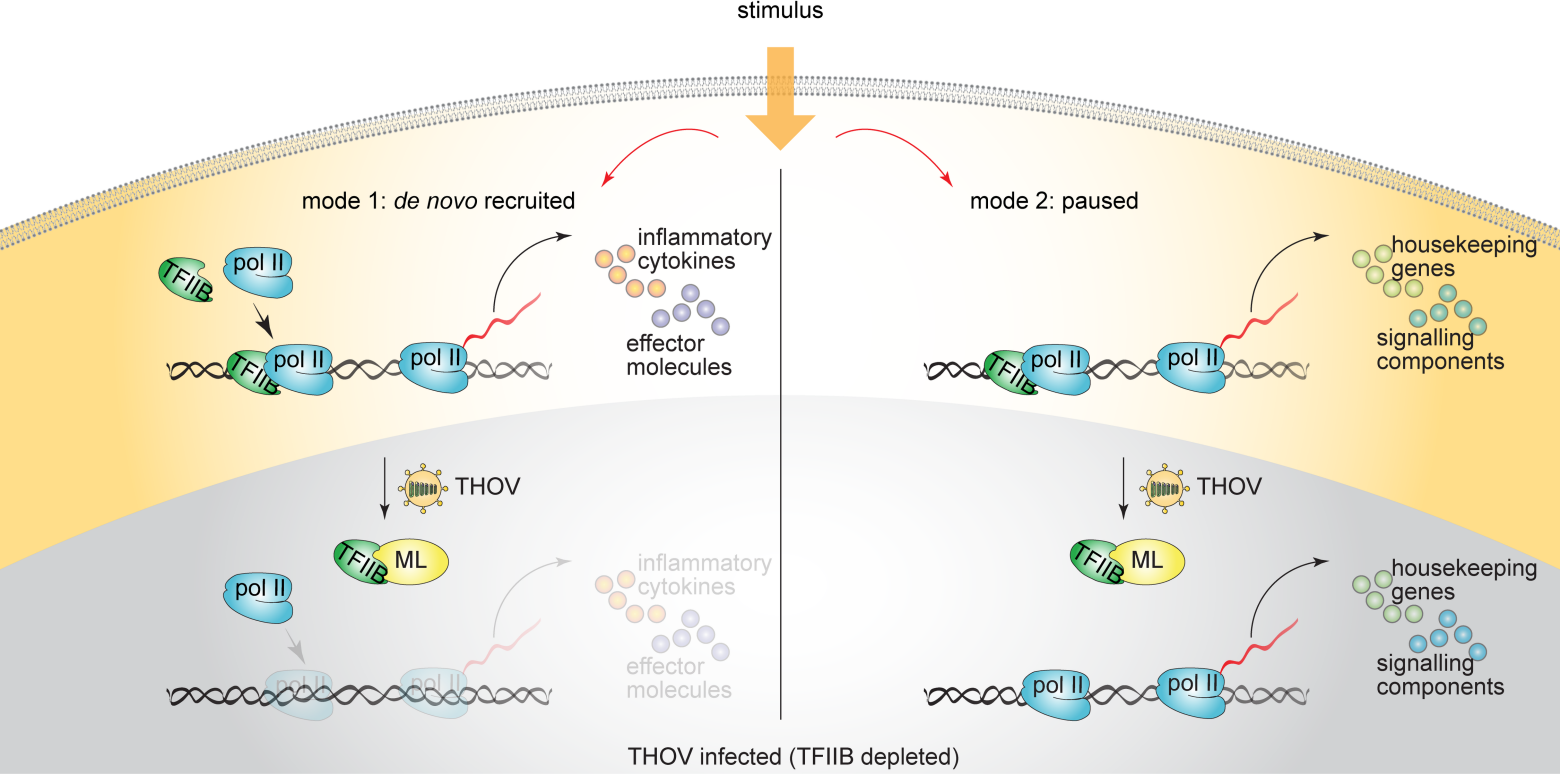
ML or TFIIB-depletion-mediated regulation of innate immune response and general transcription. When the cells are stimulated with a ligand, expression of a responsive gene can be activated by de novo recruitment of Pol II, which requires assembly of PIC (mode 1), or by releasing paused Pol II into the gene body (mode 2). In the presence of ML or after TFIIB depletion, expression of genes regulated by mode 1 (mostly cytokines and antiviral effector molecules) is severely impaired, while genes regulated by mode 2 (signalling components and housekeeping genes) continue to be expressed normally.

We generated GFP-fusion constructs containing C-terminal fragments of ML (Fig 7A) and tested their ability to directly associate with TFIIB in co-precipitation experiments with FLAG-TFIIB (Figs 7B and S7A). The 38 aa bearing GFP-ML (266c) protein failed to bind TFIIB, while GFP-ML (257c; C-terminal 47 aa), GFP-ML (247c; C-terminal 57 aa) and GFP-ML (247–294) successfully precipitated with TFIIB (Figs 7B and S7A). This suggested that binding of TFIIB requires the C-terminal “L” portion of ML as well as additional amino acids in the C-terminus of M. To assess their functionality, we co-transfected the same fragments with ISRE reporter into HEK293 cells (Fig 7B bottom panel). After stimulation with IFN-α, only the 57 aa ML fragment (GFP-ML (247c) was able to inhibit ISRE promoter activation, but not EF1-α promoter (Figs 7B and S7B). ML (257c) and ML (247–294) were able to interact with TFIIB, but failed to inhibit ISRE activation, indicating that the C-terminal 47 to 57 aa are required for the functional inhibition of TFIIB. To further narrow down the active region, we generated 2 aa truncation mutants, fused to mCherry (Fig 7A) and tested their ability to bind TFIIB and to block the activation of ISRE reporter upon IFN-α stimulation (Fig 7C). Only ML fragments (247c) and (249c) were able to inhibit ISRE induction, but not EF1-α promoter activity (Fig 7C bottom panel, S7C Fig). We concluded that the inhibitory domain of ML is thus located to aa 249–304 and consists of the L-region and a minimal part of M (miniM)–thus here called the miniML domain. Mimicking THOV and using its miniML inhibitory domain to target a single transcription factor TFIIB, one could modulate a wide variety of pro-inflammatory cytokines and effector molecules requiring *de novo* recruitment of the polymerase complex without affecting general gene expression and genes required for cellular homeostasis, growth, maintenance of protein stability, energy metabolism and signalling in particular (Fig 8).

## Discussion

The innate immune system is comprised of a functional network between cytokines and antiviral effector molecules and constitutes an essential antiviral defence barrier. Here, we identified a mechanism that involves viral targeting of a single general transcription factor, TFIIB, and allows perturbance of the innate immune system at a broad scale with minimal influence on other ongoing transcriptional processes. Through alternative splicing, THOV expresses the immunoregulatory Matrix protein variant ML, that bears a 38 amino acid C-terminal extension as compared to M. Unbiased AP-MS analysis shows that ML binds two proteins, TFIIB and CAPN15. However, the inhibitory properties of ML rely solely on the ability to bind TFIIB, while CAPN15 appears to be dispensable for immunoregulation on transcriptional level. In contrast to other viruses that degrade their target proteins [34,35], THOV does not lead to destabilisation of TFIIB or any other protein of the PIC. Rather, ML generates a TFIIB depleted environment in the nuclei of infected cells by re-localizing TFIIB into the cytoplasm. In this way, TFIIB would not be available for transcriptional purposes and won’t be able to undergo regulatory post-translational modifications, such as phosphorylation on Ser65 [51], required for transcription initiation. The ability to translocate TFIIB, may be explained by the fusion of the TFIIB-interaction region (miniML) to the matrix protein M. Orthomyxoviruses replicate and partially assemble in the nucleus before the translocation process into the cytoplasm, in case of THOV initiated by the accumulation of M in the nucleus. Given the limited coding capacity of THOV, fusion of miniML to M may provide the virus with a possibility to shuttle ML between the nucleus and the cytoplasm along with its target TFIIB. In addition, the miniML on its own is hydrophobic, resulting in limited solubility and stability. From a virus perspective, fusion to the M protein may therefore be beneficial to increase functionality of miniML.

While TFIIB is a general transcription factor and considered to be essential for the transcription of all known genes [5], wt THOV infection did not affect transcription of the majority of genes but showed prominent inhibition of IFN and ISG expression. This effect had been previously attributed to specific inhibition of the transcription factor IRF3 [38]. However, transcriptome-wide assessment of gene expression in infected cells, as well as reporter assays studying gene regulation at the promoter level, clearly show that ML has activity on inducible gene expression that goes beyond IRF3 inhibition. Intriguingly, in contrast to inflammation-related promoters, the activity of IRF1 and HSP70 promoters as well as expression of EGF target genes were not affected by ML presence. Remarkably, a large proportion of genes responsive to EGF are regulated by polymerase pausing, i.e. show deposited Pol II at their transcription initiation sites prior to their activation [46,52,53], a property that has also been shown on reporter constructs coding for isolated promoters [17]. Interestingly, IRF3 targets have been reported to mostly require *de novo* recruitment of Pol II, while RELA targets can be regulated by both, polymerase pausing or *de novo* recruitment of Pol II [48]. Our experiments suggest that promoters, which are known to be regulated by polymerase pausing are not affected by ML, while promoters that rely on *de novo* recruitment of polymerase to the transcriptional start site are sensitive to ML presence. The apparent specificity of IFN inhibition in the context of virus infection, is not mediated by ML itself but results from the nature of the given experiment: virus infection activates an IRF3 dependent type-I interferon response that requires *de novo* recruitment of Pol II to transcriptional start sites. ML inhibits this recruitment, resulting in an apparent block of interferon gene induction. However, combining other stimuli with ML expression would result in a similar specific pattern, given the recruitment of Pol II is required for expression of the induced genes.

These data indicate that ML is inhibiting *de novo* recruitment of polymerase to transcriptional start sites and that TFIIB plays a predominant role in this process, while it is dispensable to regulate expression from pre-loaded promoters, at least after Pol II has been associated. Experimental depletion of TFIIB phenocopied ML activity. Stimulation experiments using IFN-α, TNF-α or EGF showed that TFIIB depletion allowed to discriminate between two separate populations of response genes: some genes were induced normally despite TFIIB knockdown (non-affected) while other genes were severely impaired in expression in the absence of TFIIB (affected). This behaviour could be explained by Pol II and TFIIB occupancies at their promoters: under steady-state conditions non-affected genes had prominent Pol II and TFIIB levels loaded in the promoter region (paused polymerase). The affected group, however, displayed significantly lower Pol II and TFIIB loading, and their expression would therefore require *de novo* recruitment of the transcriptional apparatus. It has been reported that TFIIB is dispensable for the activation of some genes [45,54] and that TFIIB might be regulating only specialized versus housekeeping genes during cardiac hypertrophy [55]. It seems surprising that TFIIB depletion in mammalian cells shows no effect on cell viability and general gene or protein expression. However, the strict requirement of TFIIB has mostly been established through the excellent work studying *de novo* recruitment of Pol II using *in vitro* reconstitution systems employing recombinant proteins or in lower eukaryotes (*Saccharomyces cerevisiae*), which do not use polymerase pausing as gene regulation mechanism [56–60] and may thus be more dependent on TFIIB for general transcriptional processes. Regulation of TFIIB levels in higher eukaryotes may allow an additional layer of gene expression control, for instance, during development [61], injury [62], and cancer [63], which is in line with the high mobility and short chromatin association time of TFIIB as compared to Pol II [64–68]. Transient association of TFIIB with chromatin [68] may be sufficient to place Pol II at the transcriptional start site. Even if TFIIB is dissociated, depleted or sequestered by viruses, genes that bear loaded Pol II on their promoters can still be transcribed. In contrast, genes that are not loaded with Pol II require *de novo* recruitment and are therefore dependent on TFIIB activity.

Among genes that are highly dependent on *de novo* recruitment of Pol II are many inflammatory cytokines and antiviral effector molecules. In contrast, housekeeping genes and signalling components commonly show paused promoters, which gives them an advantage to bypass complete assembly of the transcriptional apparatus and allows them to react instantly to incoming stimuli. This differential mode of transcriptional regulation appears to be used for expression of immune-related genes: while many cytokines and chemokines mounting a pro-inflammatory environment require *de novo* recruitment of Pol II, negative regulators commonly show paused promoters [69]. Such regulation may allow the immune system to keep at bay undesired inflammation and have an easy access to negative regulators. Indeed, depletion of TFIIB or delivery of ML impairs expression of essential pro-inflammatory molecules (CXCL10, IL-6, IL-8, IFITs etc.), while signalling components (IRFs, IκBα, SMAD3, BCL6 etc.) and negative regulators of inflammation (A20, TGF-β) or genes required for tissue repair (ITGA1, ITGA2, PLAUR, EPHA2 etc.) are unaffected. Intriguingly, while genes requiring *de novo* Pol II recruitment show different Pol II occupancy depending on the cell type [70,71], paused genes generally have lower cell-to-cell expression variability [72].

Given its regulatory properties, targeting of TFIIB by viruses is very beneficial for the successful infection: while expression of pro-inflammatory cytokines that are required to mount an antiviral response are inhibited, negative regulators which mediate a tolerogenic environment as well as tissue repair factors are expressed (Fig 8). Importantly, modulation of TFIIB activity provides an exciting opportunity to therapeutically control selected gene expression without massively affecting global transcription. Application of miniML, an active sequence of the ML-TFIIB interacting domain, mimics the viral activity and therefore allows to modulate gene expression depending on the context. Employment of miniML may allow therapeutic taming of overshooting immune responses and could therefore be beneficial to treat diverse immunopathologies, such as septic shock, specific pathogen-induced immunopathologies, autoimmunity as well as antiviral immunity. Furthermore, gene expression of most dsDNA viruses [45,73–75] requires the host apparatus including *de novo* recruitment of the polymerase complex, thus, interference with TFIIB activity may allow to modulate their viral gene expression and viral spread.

## Materials and methods

### Reagents, cell lines and viruses

Recombinant human interferon-α (IFN-α) was a kind gift from Peter Stäheli, recombinant human TNF-α and IFN-γ were purchased from PeproTech, recombinant human EGF was a kind gift from Kirti Sharma, PMA from Felix Meissner, NiCl_2_ was purchased from Sigma-Aldrich. HEK293T transgenic for the Mx1-promoter driven firefly luciferase gene were described previously [76], HeLa FlpIn were a kind gift from Andrea Musacchio, HeLa S3 (CCL-2.2) and Vero E6 (CL-1586) were purchased from ATCC, HEK293 were a kind gift from Andrew Bowie, HeLa Kyoto expressing GFP-tagged TFIIB from BAC transgene were from Ina Poser [77]. All cell lines were maintained in DMEM (GE Healthcare Life Sciences) containing 10% foetal calf serum (GE Healthcare Life Sciences) and antibiotics (100 U/ml penicillin, 100 μg/ml streptomycin). Duplex siRNAs targeting human TFIIB (siGENOME SMARTpool) or Scrambled control were from Dharmacon (GE Healthcare Life Sciences). Recombinant THOVs expressing ML (rTHOV-wt), lacking ML (rTHOV-ΔML) or bearing mutated ML proteins (rTHOV-SW and rTHOV-TE) were described previously [40,78,79]. Virus infections were performed in reduced medium volume for 1 h at 37°C with subsequent exchange of medium and incubation at 37°C for desired periods of time.

Thiazolyl blue tetrazolium bromide (MTT) was from Sigma-Aldrich. Primary antibodies used in this study were the following: GST (Cell Signaling Technology 2624), FLAG M2 (Sigma-Aldrich F3165), β-actin-HRP (Santa Cruz sc-47778), THOV NP and THOV M/ML were described previously [80], HA (Cell Signaling Technology 3724), TFIIB (Cell Signaling Technology 4169), β-tubulin (Sigma-Aldrich), histone H3 (Abcam), GFP (Invitrogen A6455), mCherry (Rockland 600-401-P16), GFP-DyLight-488 (Rockland 600-141-215). Secondary antibodies detecting mouse and rabbit IgG were from Jackson ImmunoResearch and Dako. DAPI and secondary antibodies for immunofluorescence were purchased from Invitrogen.

### Plasmids and constructs

pCAGGS expression plasmids for HA- and GST-tagged M and ML and FLAG-tagged TFIIB were described previously [40,78]. pCAGGS-eGFP-FLAG was a kind gift from Urs Schneider, pCAGGS-FLAG-CAPN15 was generated by inserting human CAPN15 into pCAGGS. GFP, GFP-ML(266c), GFP-ML(257c), GFP-ML(247c), GFP-ML(247–294) were generated by inserting GFP and ML fragments into pcDNA3. ML fragments 247c to 257c were generated by site-directed mutagenesis and inserted into pmCherry. pGEX-GST-TFIIB was described previously [81]. Primers can be provided upon request. The following reporter constructs were used in this study: pISRE-luc was purchased from Stratagene, pGL3-Mx1-ff-luc was described previously [82], NFkB-luc was a kind gift from Andrew Bowie, NFAT-luc and ISG54-luc from Tilmann Bürckstümmer, EF1-α-ren from Engin Gürlevik, pIRF1-GAS-ff-luc was described previously [40], HSP70-luc was a kind gift from Mark Hipp.

### IFN bioassay and reporter assays

Total amounts of IFN-α/β in cell supernatants were measured by using 293T cells stably expressing the firefly luciferase gene under the control of the mouse Mx1 promoter (Mx1-luc reporter cells) [76]. Briefly, cell supernatants were harvested and virus particles were removed with Amicon spin columns with a cutoff of 100 kDa (Millipore) according to the manufacturer's instructions. Mx1-luc reporter cells were seeded into 96-well plates in sextuplicates and were treated 24 hours later with filtered supernatants diluted 1:10 in DMEM-5% FCS. At 16 hours post incubation, cells were lysed in passive lysis buffer (Promega), and luminescence was measured with a microplate reader (Tecan). The assay sensitivity was determined by a standard curve.

For reporter assays, HEK293 cells were plated in 96-well plates 24 hours prior to transfection. Firefly reporter and Renilla transfection control were transfected using polyethylenimine (PEI, Polysciences) in sextuplicates for untreated and treated conditions. In 24 hours cells were stimulated for 16 hours with corresponding inducer and harvested in passive lysis buffer (Promega). Luminescence of Firefly and Renilla luciferases was measured using dual-luciferase-reporter assay (Promega) according to the manufacturer’s instructions in a microplate reader (Tecan).

### Immunofluorescence assay

For immunofluorescence analysis, HeLa Kyoto cells stably expressing GFP-TFIIB were grown on coverslips, transfected with M/ML proteins or infected with rTHOV, and fixed with 4% /w/v) paraformaldehyde (PFA) for 15 min at room temperature, blocked in blocking buffer (1xPBS containing 0.1% foetal calf serum (w/v) and 0.1% Triton X-100 (v/v)) for 1 hour at room temperature. Stainings were performed for 1 hour at room temperature in blocking buffer. Confocal imaging was performed using an LSM780 confocal laser scanning microscope (ZEISS) equipped with a Plan-APO 63x/NA1.46 immersion oil objective (ZEISS).

### Cellular fractionation

For cytoplasmic-nuclear fractionation, HEK293T cells were seeded in 9 cm dishes, transfected with HA-tagged M/ML and FLAG-TFIIB proteins. After 24 hours the cells were washed and fractionated in sucrose-NP-40 buffer supplemented with protease inhibitors (10 mM HEPES pH 7.9, 0.34 M sucrose, 3 mM CaCl2, 3 mM MgAc, 0.1 mM EDTA, 0.5% NP-40). The lysates were incubated 10 min on ice and centrifuged at 3500 rpm for 5 min. Cytoplasm fraction (supernatant) was transferred into a new tube and mixed with 4x-Laemmli buffer. Nuclear fraction (pellet) was washed in sucrose buffer without NP-40 and resuspended in sucrose buffer with benzonase, incubated for 10 min on ice and centrifuged at 3500 rpm for 5 min. Pellets were resuspended in 1x Laemmli buffer. Both fractions were loaded on 12% SDS gel, proteins of interest were detected by western blotting.

### GST pulldown

GST and GST-TFIIB were purified from BL21 and bound to GST-agarose beads for 2 h at 4°C. Expression and purity was controlled by SDS-PAGE and Coomassie staining. GFP-ML fragments were *in vitro* transcribed and translated (IVT) using TNT quick coupled transcription/translation kit (Promega) and radioactively labelled with [^35^S]-Methionine/Cysteine. GST-fusion proteins were incubated with IVT fragments for 2 h at 4°C, washed, separated on 12% SDS-gel and detected by autoradiography (Kodak BiomaxMR).

### Affinity purification and quantitative LC-MS/MS

For affinity purification, cell lysates were prepared by lysing HEK293 cells expressing HA- or GST-tagged M/ML proteins and FLAG-tagged CAPN15 or TFIIB for 30 min on ice in TAP lysis buffer (50 mM Tris pH 7.5, 100 mM NaCl, 5% (v/v) glycerol, 0.2% (v/v) Nonidet-P40, 1.5 mM MgCl_2_ and protease inhibitor cocktail (EDTA-free, cOmplete; Roche)). For affinity-purification with HA-tagged proteins, HA affinity resin (Sigma-Aldrich) was incubated with cell lysate in TAP lysis buffer for 60 min at 4°C on a rotary wheel. For affinity-purification with Flag-M2-tagged proteins, Flag-M2 affinity resin (Sigma-Aldrich) was incubated with cell lysate and processed as above. Beads were washed three times with TAP lysis buffer, followed by two times with TAP wash buffer [lacking 0.2% (v/v) Nonidet-P40], boiled in 2x Cell Signaling SDS buffer for 5 min at 95°C and subjected to SDS-PAGE and Western Blot analysis.

For quantitative purification of ML-binding proteins, HA affinity resin (Sigma-Aldrich) was incubated with lysates of HEK293 cells expressing HA-tagged M or ML proteins and processed as above. Four independent affinity purifications were performed for each protein. Bound proteins were denatured by incubation in 6 M urea-2 M thiourea with 1 mM DTT (Sigma-Aldrich) for 30 min and alkylated with 5.5 mM iodoacetamide (Sigma-Aldrich) for 20 min. After digestion with 1 μg LysC (WAKO Chemicals USA) at room temperature for 4 h, the suspension was diluted in 50 mM ammonium bicarbonate buffer (pH 8). Protein solution was digested with trypsin (Promega) overnight at room temperature. Peptides were purified on stage tips with three C18 Empore filter discs (3M) and analysed by mass spectrometry as described previously [83]. Briefly, peptides were eluted from stage tips and separated on a C18 reversed-phase column (Reprosil-Pur 120 C18-AQ, 3 μM, 150×0.075 mm; Dr. Maisch) by applying a 5% to 30% acetonitrile gradient in 0.5% acetic acid at a flow rate of 250 nl/min over a period of 95 min, using an EASY-nanoLC system (Proxeon Biosystems). The nanoLC system was directly coupled to the electrospray ion source of an LTQ-Orbitrap XL mass spectrometer (Thermo Fisher Scientific) operated in a data dependent mode with a full scan in the Orbitrap cell at a resolution of 60,000 with concomitant isolation and fragmentation of the ten most abundant ions in the linear ion trap.

### Pulsed SILAC and mass spectrometry

HeLa cells were grown in normal medium containing light (L) amino acids and infected with rTHOV viruses for 1 h. After 18 hours the cells were incubated in starvation medium (lacking Lys and Arg) for 30 min. Subsequently, SILAC medium containing heavy (H) labelled amino acids (Lys8, Arg10) was added. 6 hours later total protein lysates were prepared and subjected to LC-MS/MS analysis. Briefly, lysates were prepared in SDS lysis buffer (50 mM Tris pH 7.5, 4% sodium dodecyl sulfate), boiled for 5 min at 95°C, sonicated for 15 min with a Bioruptor (Diagenode) and centrifuged for 5 min at 16,000× *g* at room temperature. Protein concentration was determined by Lowry assay (DC Protein Assay, BioRAD), and 50-μg aliquots were reduced with 10 mM DTT for 30 min, alkylated with 55 mM IAA for 20 min at room temperature, and precipitated with 80% acetone for 3 h at 20°C. After centrifugation for 15 min at 16,000× *g* at 4°C, pellets were washed with 80% acetone, dried for 30 min at room temperature and dissolved in 6 M urea-2 M thiourea. Proteins were digested with LysC and trypsin at room temperature and peptides were purified on stage tips and analysed by LC-MS/MS using an Easy nano LC system coupled to a Q Exactive mass spectrometer (Thermo Fisher Scientific). Peptide separation was achieved on a C18-reversed phase column (Reprosil-Pur 120 C18-AQ, 1.9 μM, 200×0.075 mm; Dr. Maisch) using a 95-min linear gradient of 2 to 30% acetonitrile in 0.1% formic acid. The mass spectrometer was set up to run a Top10 method, with a full scan followed by isolation, HCD fragmentation and detection of the ten most abundant ions per scan in the Orbitrap cell.

### Quantitative analysis of RNA synthesis in virus-infected cells

Vero cells were seeded in 6-well plates, infected with rTHOV viruses at high MOI and subjected to metabolic labelling of newly synthesized RNA at different time points after infection. For labelling, the cells were incubated in [^3^H]-5-Uridine-containing medium (20 μCi/ml und 1 ml/well) for 1 h and lysed in the lysis buffer from RNA extraction kit with subsequent total RNA extraction (RNeasy, Qiagen).

### Cell viability assay

Cell viability was determined by MTT assay. Briefly, 0.5 mg/mL MTT were added to the cells and incubated for 3 h at 37°C, reaction was stopped by aspirating the medium and solving the crystals in 1:1 mix of DMSO:ethanol for 15 min shaking at room temperature, followed by absorbance (570 nm) measurement using a microplate reader (Tecan).

### siRNA-mediated knockdown

Duplex siRNAs (100 pmol of siRNA per 1×10^5^ cells) were transfected using Neon Transfection System (Invitrogen) according to the manufacturer´s instructions for HeLa cells.

### Western blot

Cells were lysed in 1x SDS lysis buffer (62.5 mM Tris HCl pH 6.8, 2% SDS, 10% glycerol, 50 mM DTT, 0.01% bromophenol blue) containing protease inhibitors. Protein lysates were boiled at 95°C for 5 min, separated by SDS-PAGE and transferred onto nitrocellulose membrane (GE Healthcare Life Sciences). After blocking in 1xPBS containing 5% nonfat dry milk (Sigma-Aldrich) and 0.05% Tween for 30 min at room temperature, the membrane was first incubated for 1 h at room temperature with primary antibodies and then washed three times in 1x PBS containing 0.05% Tween with subsequent incubation in horseradish peroxidase-conjugated secondary antibodies and three additional washes. Detection was performed with SuperSignal West Femto kit (Pierce).

### RT-qPCR

Total RNA was isolated using the NucleoSpin RNA II kit (Macherey-Nagel), including on-column DNase digestion, and 200 to 500 ng of RNA was reverse transcribed with the PrimeScript RT Master Mix (Takara). RNA levels were then quantified by real-time RT-PCR using the QuantiTect SYBR Green RT-PCR kit (Qiagen) and a CFX96 Touch Real-Time PCR Detection System (BioRad). Each cycle consisted of 15 sec at 95°C, 30 sec at 50°C and 30 sec at 72°C, followed by melting curve analysis. Primer sequences are provided in S8 Table.

### Transcriptome analysis

Total RNA was isolated as described above. Library preparation and RNA sequencing was performed by Max Planck-Genome-Centre Cologne, Germany (http://mpgc.mpipz.mpg.de/home/). For RNA-Seq analysis trimmed and quality-filtered reads were mapped with Tophat2 [84] to the Ensembl genome annotation (version 70) and the human genome assembly GRCh37. Expression levels and differential gene expression were quantified using the cufflinks2 package [85]. In order to stabilize extreme fold change ratios generated by cuffdiff, we filtered out genes with a maximum per sample read count below 10 and calculated stabilized fold change values as the ratio of sample FPKM values each shifted by +2. The datasets can be found under GEO GSE105154.

### Bioinformatic analyses

Raw mass-spectrometry data were processed with MaxQuant software versions 1.4.1.8 and version 1.5.1.6 [86] using the built-in Andromeda search engine to search against human and mouse proteomes (UniprotKB, release 2012_06) containing forward and reverse sequences, and the label-free quantitation algorithm as described previously [83,87]. In MaxQuant, carbamidomethylation was set as fixed and methionine oxidation and N-acetylation as variable modifications, using an initial mass tolerance of 6 ppm for the precursor ion and 0.5 Da for the fragment ions. For SILAC samples, multiplicity was set to 2 and Arg10 and Lys8 were set as heavy label parameters. Search results were filtered with a false discovery rate (FDR) of 0.01 for peptide and protein identifications. Protein tables were filtered to eliminate the identifications from the reverse database and common contaminants. Data were analysed in Perseus.

In analysing mass spectrometry data from affinity purifications, only proteins identified on the basis of at least two peptides and a minimum of three quantitation events in at least one experimental group were considered. Label-free quantitation (LFQ) protein intensity values were log-transformed and missing values filled by imputation with random numbers drawn from a normal distribution, whose mean and standard deviation were chosen to best simulate low abundance values. Significant interactors of bait proteins were determined by multiple equal variance t-tests with permutation-based false discovery rate statistics. We performed 250 permutations and the FDR threshold was set between 0.02 and 0.1. The parameter *S*_0_ was empirically set between 0.2 and 1, to separate background from specifically enriched interactors.

For data analysis from pulsed SILAC experiments, we used log-transformed heavy to light protein ratios. Only proteins with valid values were considered for analysis. Profile plots were generated using LFQ intensities of log-transformed heavy-labelled protein intensities. Missing values were filled by imputation.

### ChIP-Seq data extraction and analyses

For the quantification of occupancies of TFIIB, Pol II, NELF and DSIF, we re-analysed published HeLa ChIP-Seq data from [49,50]. Trimmed and quality-filtered reads were mapped to genome assembly GRCh37 with bowtie2 [88] and filtered by mapping quality score (cutoff 30). A custom genome annotation file was generated based on Ensembl canonical transcripts containing target intervals relative to the transcription start site of -500 to +500bp (promoter), -50 to +300bp (downstream promoter) and +300bp to transcript end (gene body). Transcripts shorter than 1000bp were not considered. Read-coverage for these intervals was quantified using featureCounts [89] (TFIIB and H3K4me3: promoter; Pol II, NELF and DSIF: downstream promoter; Pol2: gene body). Where replicate samples were available, counts were normalized by library size, background-corrected by subtraction of input control and averaged across replicates. Gene pausing indices were calculated based on the Pol II samples from [50] as the length-normalized count ratio between the downstream promoter and the gene body intervals [12,72].

P-values for the significance of differential read coverage between selected sets of genes were calculated using a negative binomial count model with a log link function in R (using the MASS package).

## Supporting information

S1 Fig A) Heat map of proteins enriched in AP-MS analysis of M and ML precipitates. B) IP of FLAG-tagged CAPN15 and TFIIB (and GFP as a control) and co-IP of ML during virus infection (FLAG-TFIIB and FLAG-CAPN15 were transiently overexpressed in HEK293 cells, which were then infected with THOV wt, dML or mut at MOI 5 for 24 hours.
(TIF)

S2 Fig A) Confocal immunofluorescence analysis of HeLa Kyoto cells stably expressing GFP-TFIIB and transiently transfected with HA-M or HA-ML for 24 hours. HeLa cells were treated as indicated, fixed and stained with GFP-DyLight488, HA+msAlexa594 and DAPI and subjected to confocal microscopy. B) Schematic representation of pulse SILAC experiment. HeLa cells were infected with THOV wt or ΔML, after 18 hours, the cells were starved in medium lacking Lys and Arg for 30 min and then pulsed with SILAC medium containing heavy Lys8 and Arg10 for another 6 hours. Cells were harvested and subjected to the whole proteome LC-MS/MS analysis. C) Effect of ML presence on the levels of polymerase II subunits and general transcription factors after THOV wt or ΔML infection. Presented are log2-transformed LFQ intensities.</
SI_Caption>
(TIF)

S3 Fig A) Schematic representation of transcriptome analysis. HeLa S3 cells were infected with THOV-wt, THOV-DML, THOV-SW or left uninfected for 16 hours. Total RNA was extracted, and after polyA enrichment samples were submitted for RNA-seq analysis. B) Heat map of hierarchically clustered log2 FPKM values normalized by subtracting median of changing genes identified by transcriptome analysis (RNA-seq). C) Polar charts representing enriched transcription factors and numbers of target genes, identified by upstream regulator analysis of enriched cluster from (B) (genes induced by THOV-ΔML and THOV-SW) (iRegulon).
(TIF)

S4 Fig A-C) Western blot analysis of HEK293 cells transfected with reporters and GST-tagged M, ML and ML(SW). D) Western blot analysis of HeLa-FlpIn cells expressing HA-tagged ML before and after doxycycline induction.
(TIF)

S5 Fig A) Schematic representation of transcriptome analysis of HeLa cells before and after TFIIB knockdown and TNF-α treatment. HeLa S3 cells were electroporated with indicated siRNAs. In 24 hours they were stimulated with TNF-α (20 ng/ml) for 2 hours. Total RNA was extracted, polyA enriched and submitted for RNAseq analysis. B) qPCR analysis of 3 independent replicates used for RNAseq analysis. (ND) not detected. C) Western blot analysis of HeLa cells transfected with Scrambled or TFIIB-targeting siRNA and mock-infected or THOV-ΔML infected for 16 hours.
(TIF)

S6 Fig A) Schematic representation of de novo recruited, paused and elongating Pol II. B) Features of Pol II, NELF, DSIF and TFIIB occupancy at the promoter and in the gene body used to discriminate de novo recruitment, paused and elongating Pol II.
(TIF)

S7 Fig A) GST pulldown of GST or GST-TFIIB and radioactively labelled in vitro translated GFP-ML fragments. B) Reporter assay in HEK293 cells, where Renilla luciferase under EF1-α promoter was co-transfected with GFP-ML fragments. C) Reporter assay in HEK293 cells, where Renilla luciferase under EF1-α promoter was co-transfected with mCherry-ML fragments.
(TIF)

S1 Table Interactors of THOV ML protein compared to THOV M. THOV ML-HA and THOV M-HA were overexpressed in HEK293 cells. Precipitates of HA-tagged baits were analysed by AP-MS/MS. Presented are LFQ intensities after imputation and Welch t-test comparisons of M and ML precipitates.
(XLSX)

S2 Table SILAC pulsed proteome of HeLa cells infected with THOV wt or ΔML. iBAQ_heavy—newly synthesized proteins after THOV infection; iBAQ_ratio—translation rates after THOV infection; iBAQ_complete—full (H+L) intensities of proteins after THOV infection.
(XLSX)

S3 Table Transcriptome analysis of HeLa cells infected with THOV wt, ΔML or SW. Presented are FPKM values and contrasts (fold change and q values).
(XLSX)

S4 Table Results of upstream regulator analysis. Number of genes regulated by transcription factors, identified with iRegulon as upstream regulators.
(XLSX)

S5 Table Transcriptome analysis of HeLa cells before and after TFIIB depletion and TNF-α treatment. Presented are FPKM values and contrasts (fold change and q values).
(XLSX)

S6 Table RT-qPCR data. Presented are log2 fold change values +/- error compared to untreated siScr.
(XLSX)

S7 Table ChIPseq data integration. (XLSX)

S8 Table Primer sequences used in RT-qPCR. (XLSX)
