# Supplementary figures and images for: Viral targeting of TFIIB impairs de novo polymerase II recruitment and affects antiviral immunity
Source: PLoS Pathog. 2018 Apr 30;14(4):e1006980. doi: 10.1371/journal.ppat.1006980 (PMC5927403; doi:10.1371/journal.ppat.1006980)

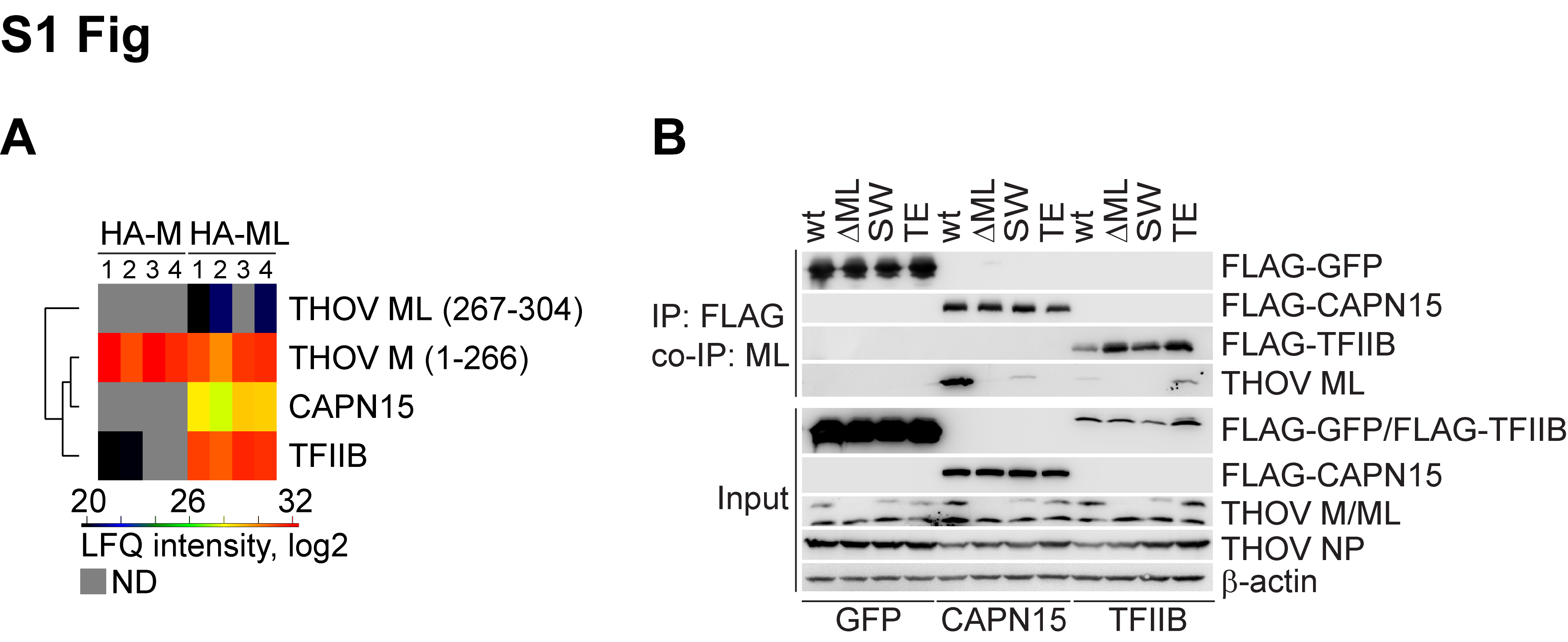

Supplement: S1 Fig — A) Heat map of proteins enriched in AP-MS analysis of M and ML precipitates. B) IP of FLAG-tagged CAPN15 and TFIIB (and GFP as a control) and co-IP of ML during virus infection (FLAG-TFIIB and FLAG-CAPN15 were transiently overexpressed in HEK293 cells, which were then infected with THOV wt, dML or mut at MOI 5 for 24 hours. (TIF) [file ppat.1006980.s001.tif]

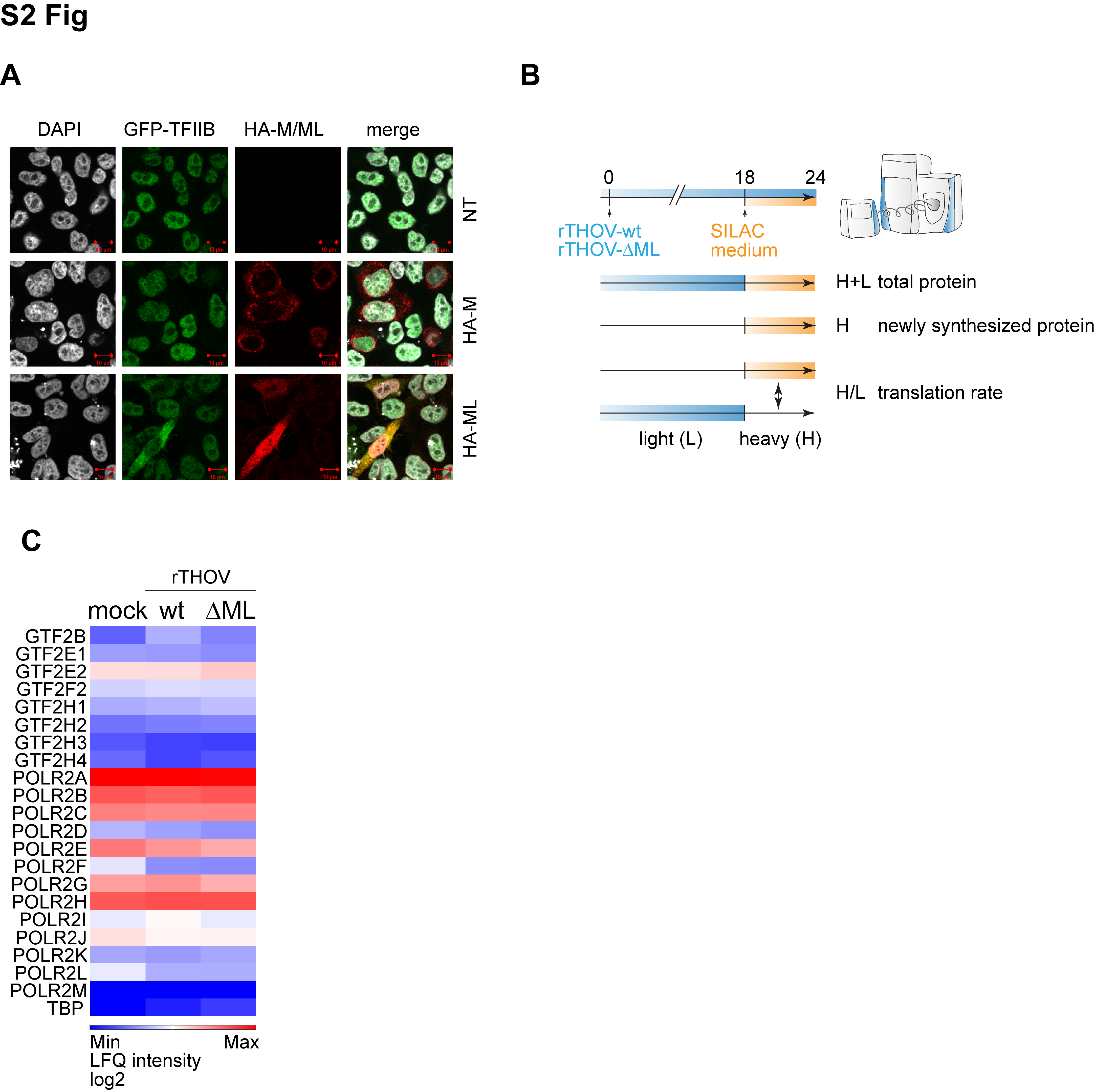

Supplement: S2 Fig — A) Confocal immunofluorescence analysis of HeLa Kyoto cells stably expressing GFP-TFIIB and transiently transfected with HA-M or HA-ML for 24 hours. HeLa cells were treated as indicated, fixed and stained with GFP-DyLight488, HA+msAlexa594 and DAPI and subjected to confocal microscopy. B) Schematic representation of pulse SILAC experiment. HeLa cells were infected with THOV wt or ΔML, after 18 hours, the cells were starved in medium lacking Lys and Arg for 30 min and then pulsed with SILAC medium containing heavy Lys8 and Arg10 for another 6 hours. Cells were harvested and subjected to the whole proteome LC-MS/MS analysis. C) Effect of ML presence on the levels of polymerase II subunits and general transcription factors after THOV wt or ΔML infection. Presented are log2-transformed LFQ intensities.</ SI_Caption> (TIF) [file ppat.1006980.s002.tif]

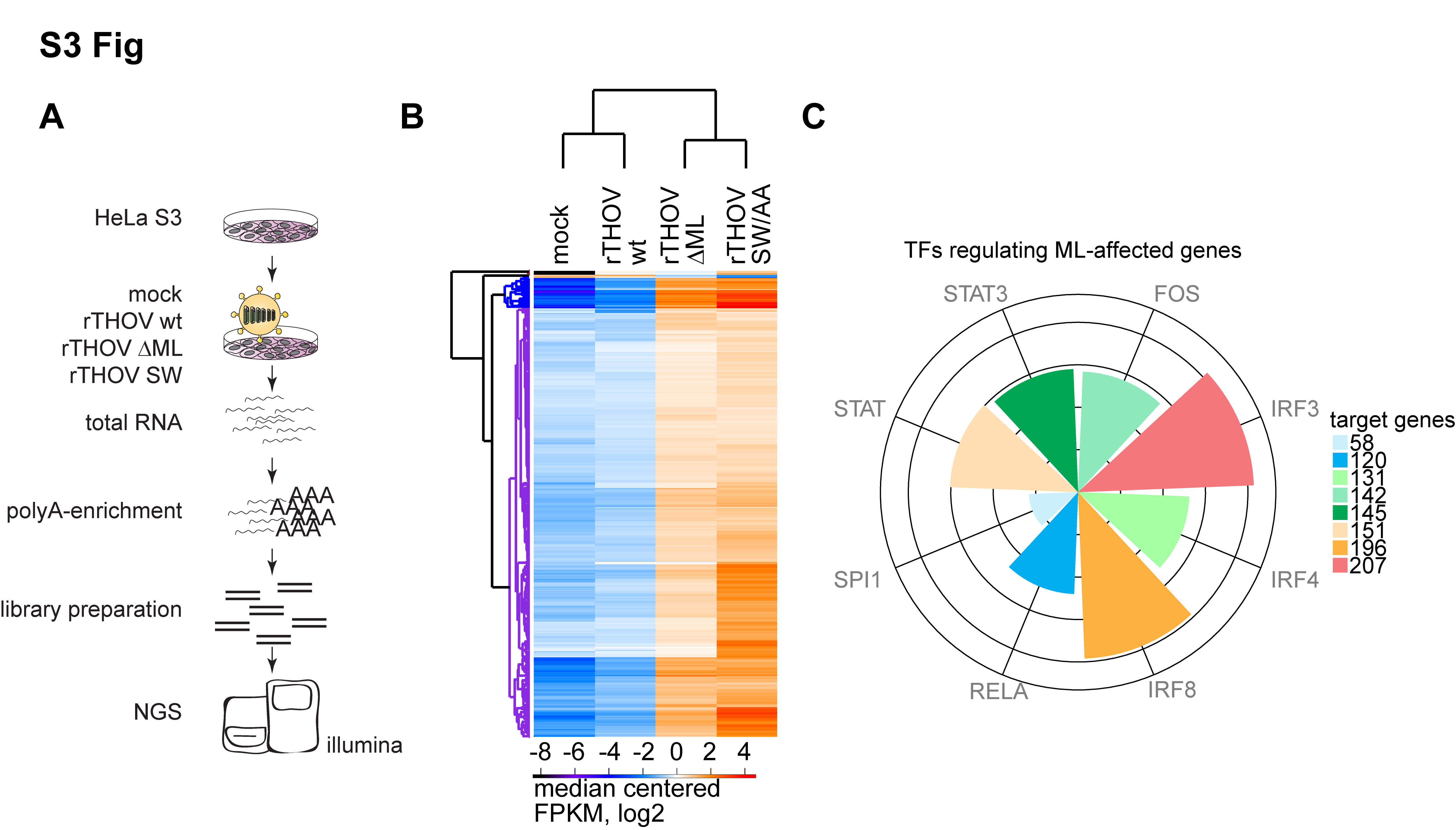

Supplement: S3 Fig — A) Schematic representation of transcriptome analysis. HeLa S3 cells were infected with THOV-wt, THOV-DML, THOV-SW or left uninfected for 16 hours. Total RNA was extracted, and after polyA enrichment samples were submitted for RNA-seq analysis. B) Heat map of hierarchically clustered log2 FPKM values normalized by subtracting median of changing genes identified by transcriptome analysis (RNA-seq). C) Polar charts representing enriched transcription factors and numbers of target genes, identified by upstream regulator analysis of enriched cluster from (B) (genes induced by THOV-ΔML and THOV-SW) (iRegulon). (TIF) [file ppat.1006980.s003.tif]

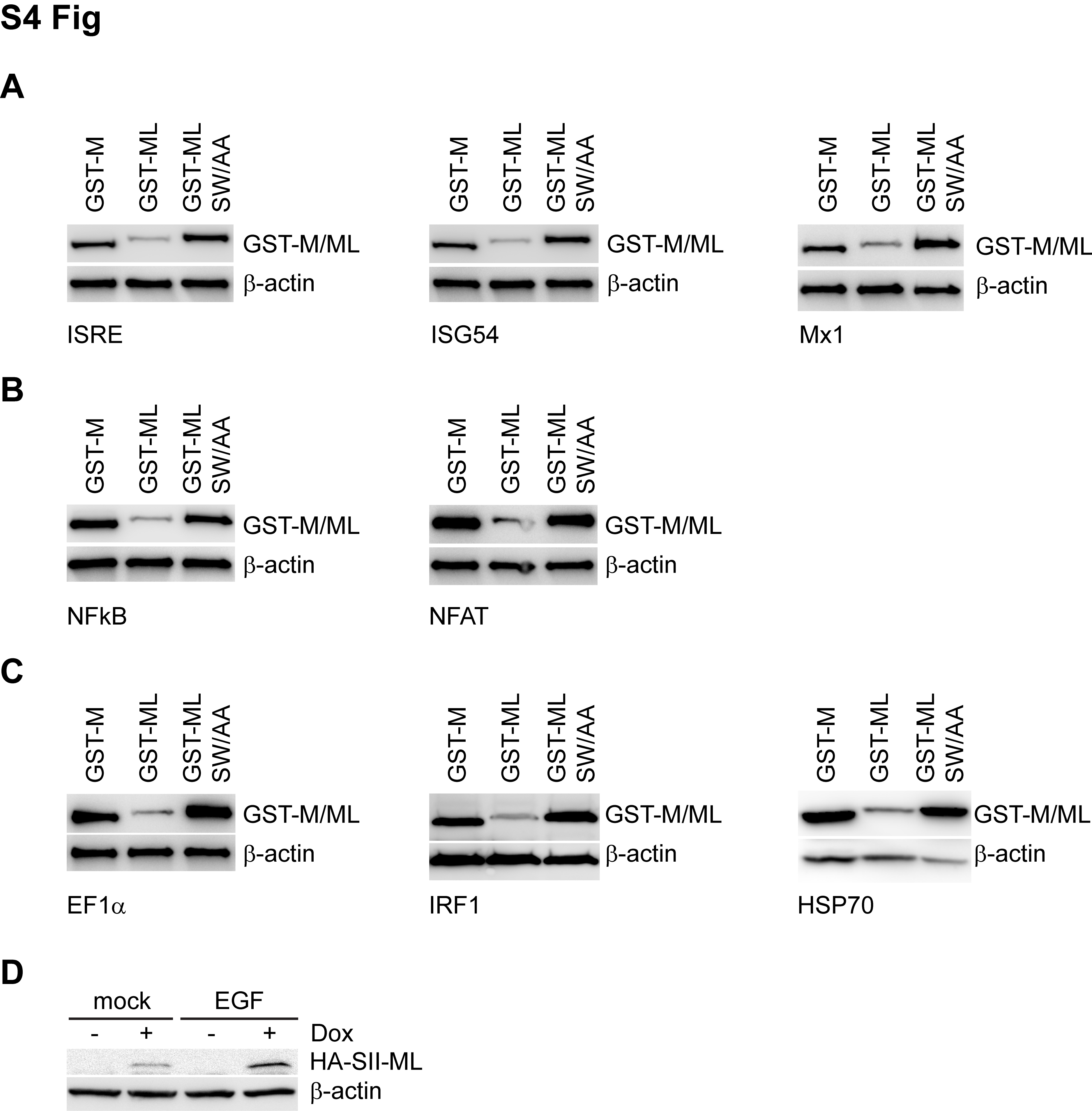

Supplement: S4 Fig — A-C) Western blot analysis of HEK293 cells transfected with reporters and GST-tagged M, ML and ML(SW). D) Western blot analysis of HeLa-FlpIn cells expressing HA-tagged ML before and after doxycycline induction. (TIF) [file ppat.1006980.s004.tif]

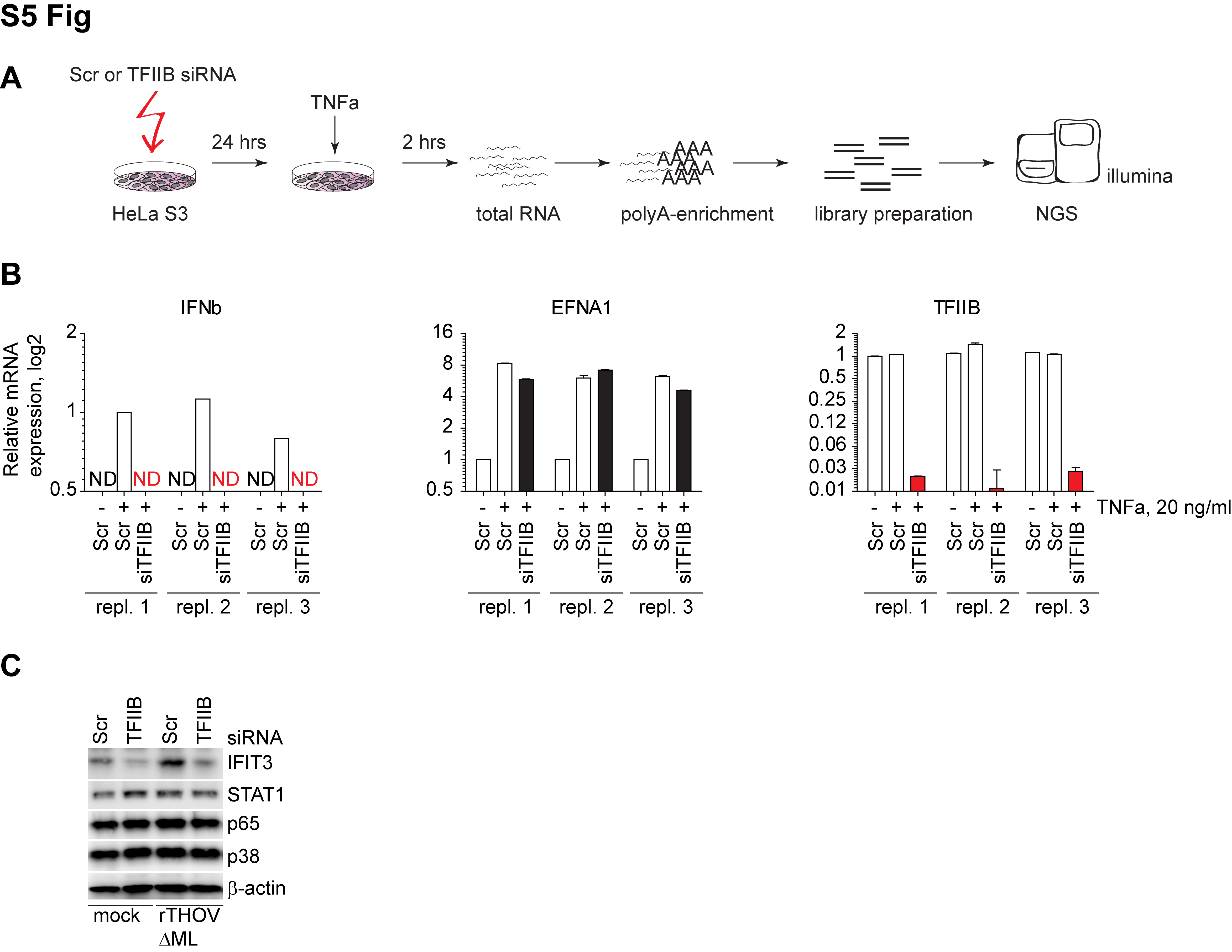

Supplement: S5 Fig — A) Schematic representation of transcriptome analysis of HeLa cells before and after TFIIB knockdown and TNF-α treatment. HeLa S3 cells were electroporated with indicated siRNAs. In 24 hours they were stimulated with TNF-α (20 ng/ml) for 2 hours. Total RNA was extracted, polyA enriched and submitted for RNAseq analysis. B) qPCR analysis of 3 independent replicates used for RNAseq analysis. (ND) not detected. C) Western blot analysis of HeLa cells transfected with Scrambled or TFIIB-targeting siRNA and mock-infected or THOV-ΔML infected for 16 hours. (TIF) [file ppat.1006980.s005.tif]

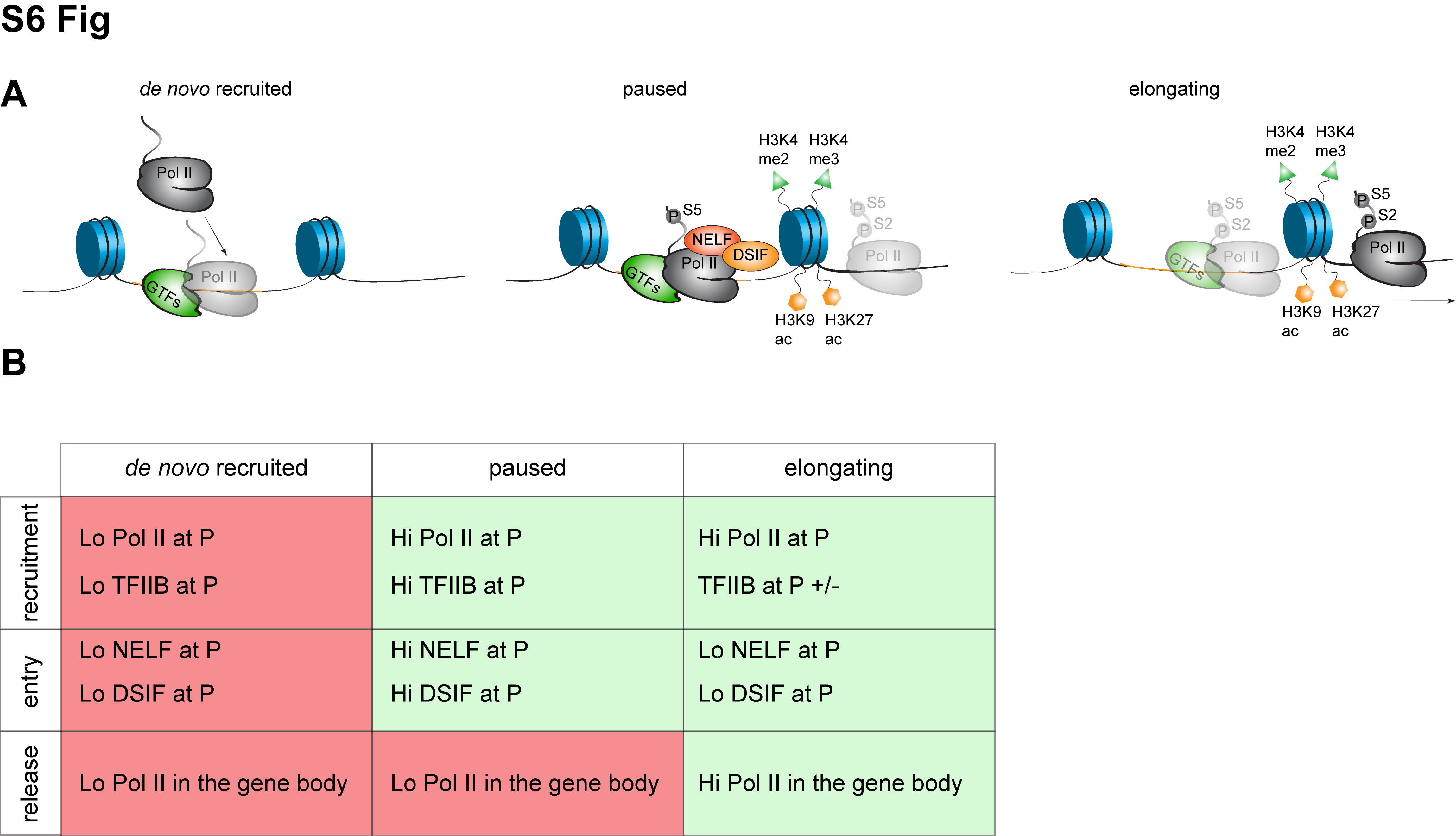

Supplement: S6 Fig — A) Schematic representation of de novo recruited, paused and elongating Pol II. B) Features of Pol II, NELF, DSIF and TFIIB occupancy at the promoter and in the gene body used to discriminate de novo recruitment, paused and elongating Pol II. (TIF) [file ppat.1006980.s006.tif]

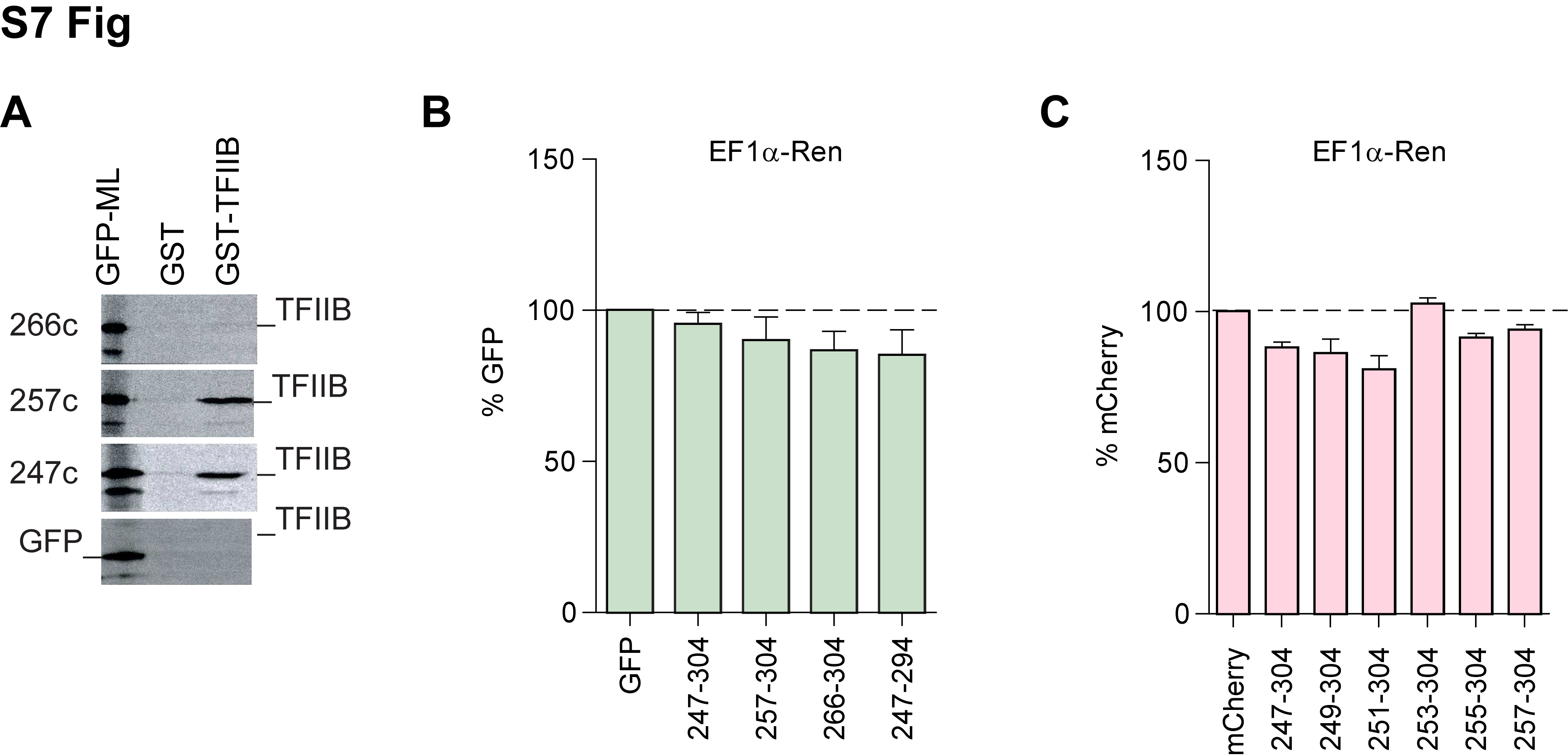

Supplement: S7 Fig — A) GST pulldown of GST or GST-TFIIB and radioactively labelled in vitro translated GFP-ML fragments. B) Reporter assay in HEK293 cells, where Renilla luciferase under EF1-α promoter was co-transfected with GFP-ML fragments. C) Reporter assay in HEK293 cells, where Renilla luciferase under EF1-α promoter was co-transfected with mCherry-ML fragments. (TIF) [file ppat.1006980.s007.tif]
